# Striatal and hippocampal contributions to instrumental learning in the mouse using high-resolution behavioral monitoring and fMRI

**DOI:** 10.1162/IMAG.a.975

**Published:** 2025-11-04

**Authors:** Eyal Bergmann, Daniela Lichtman, Admir Resulaj, Guy Yona, Ornit Nahman, Dmitry Rinberg, Itamar Kahn

**Affiliations:** Department of Neuroscience, Mortimer B. Zuckerman Mind Brain Behavior Institute, Columbia University, New York, NY, United States; Department of Neuroscience, Rappaport Faculty of Medicine, Technion—Israel Institute of Technology, Haifa, Israel; Neuroscience Institute, NYU Langone Health, New York, NY, United States; Interdepartmental Neuroscience Program, Northwestern University, Evanston, IL, United States; Center for Neural Science, New York University, New York, NY, United States; Lead contact

**Keywords:** learning and memory, goal-directed behavior, hippocampus, striatum, fMRI

## Abstract

The mammalian brain orchestrates goal-directed behavior through complex interactions between multiple memory systems, with the striatum and hippocampus playing pivotal interrelated roles. A central open question is the extent to which distinct memory signals from these systems drive learning to achieve desired goals and, once those are learned, maintain performance. Here, we used an MRI-compatible platform to obtain whole-brain functional imaging of head-fixed mice as they learn to perform a lick go/no-go odor discrimination task from the naïve state to task proficiency. Behaving mouse functional MRI (fMRI) acquired over a period of several days allowed us to characterize distributed activity as the animals learned the task, demonstrating differential involvement of the striatal and hippocampal memory systems accounting for correct and incorrect task responses. A consideration of the contribution of striatal sub-regions revealed that the responses of the dorsal striatum were correlated with improvement in reaction time, while responses in the ventral striatum were correlated with learning the task and maintaining task performance. In contrast, the dorsal hippocampus showed depressed responses to correct licks to the target odor (hits) and increased responses to incorrect licks to the non-target odor (false alarms). False alarms that were correlated with positive hippocampal responses had longer reaction times and emerged after the mouse learned the task, implicating the hippocampus in driving false memory responses. These results show that behaviorally beneficial actions were correlated with the striatum with a competing involvement of the hippocampus driving erroneous actions, setting the stage to study circuit-based mechanisms of false memory in the mouse.

## 1 Introduction

The mammalian brain supports parallel perceptual, cognitive, and control processes that subserve goal-directed behavior, the ability to integrate external and internal information to select specific actions attaining a desired outcome (Swanson, 2000). While these processes are studied in both humans and animal models, the tools and levels of investigation available are qualitatively different. Work in animals, and specifically in rodents, has focused on cellular-level measurements of neural activity during behavior and characterization of behavioral outcomes from causal circuit-level and cell-type specific manipulations (Kim et al., 2017; Sternson & Roth, 2014). In contrast, human brain research employs non-invasive macroscopic characterization of region-wide neural activity during behavior. A possible solution that can bridge the gap between human and animal studies is the use of experimental platforms that support alignment of scientific questions across communities through collection of identical types of data (Badre et al., 2015).

The need for alignment of research is especially prominent in the cognitive neuroscience of memory. In particular, explaining how striatal and hippocampal memory systems contribute to goal-directed behavior via dopaminergic signals (Shohamy & Adcock, 2010) may benefit from this approach. The striatum (Balsters et al., 2020) and hippocampus (Clark & Squire, 2013) are considered relatively evolutionary conserved, demonstrating similar form and function across species, though the extent to which the functions supported by the striatum and hippocampus are conserved is not resolved, and in particular the nature of their mutual contributions to instrumental learning is a topic of focus (Hartley & Burgess, 2005). An approach that may address gaps between findings in humans and rodents is functional MRI (fMRI), which is widely used in humans. Moreover, longitudinal fMRI interrogation of goal-directed behaviors, with task acquisition and learning occurring in the same context, will enable the identification of the contributions of multiple memory systems to associative learning (Niv, 2021).

Here, we characterized brain-wide changes that occur during olfactory-guided discrimination learning in mice. We show that mice can be trained to discriminate between olfactory stimuli in a rapid event-related go/no-go odor discrimination task, and that they all achieve task proficiency. Utilizing high-resolution behavioral monitoring, we show that learning is associated not only with task performance, but also with the adjustment of sniffing and licking patterns. Leveraging the whole-brain coverage offered by fMRI, we uncover neural responses associated with olfactory perception, motor control, and reward processing. By contrasting task conditions, we probe the contribution of the striatal and hippocampal memory systems to task performance, demonstrating differential competing involvement of these regions. Dissection of the role of different striatal sub-regions reveals specialization to motor- and reward-related processes, with the latter driving veridical memory responses. Examination of hippocampus response patterns shows that it drives false memory signals.

## 2 Methods

### 2.1 Animals

All procedures were conducted in accordance with the ethical guidelines of the National Institutes of Health and were approved by the institutional animal care and use committee (IACUC) at Technion. Ten male C57/bl6 mice (2–3 months old) were implanted with MRI-compatible head posts and housed in reversed 12 h light/dark cycle. After 7–10 days of recovery, in which mice returned to their preoperative weight, a 7–10 days water deprivation regime (1 ml/day) started. After mice weights stabilized (83.78% ± 2.75 of initial weight, mean ± SD), behavioral training began.

### 2.2 Head-post surgery

For head fixation, mice were implanted with custom-made MRI-compatible head posts, as previously described (Bergmann et al., 2016). Briefly, mice were anesthetized with isoflurane (1.5–2.5%) and received local (bupivacaine) and systemic (buprenorphine) analgesia. Then, the scalp and periosteum layer were removed to expose the skull and the head post was implanted using dental cement (Metabond, Parkell). In some cases, another layer of dental cement (Paladur, Heraeus Kulzer) was applied to improve homogeneity and reduce susceptibility artifacts. After surgery, mice received daily buprenorphine injections for 3 days.

### 2.3 Behavioral training

After post-operative recovery and water deprivation, behavioral training started with 3–7 daily lick training sessions, in which mice learned to lick the water port for rewards (Supplementary Fig. S1). After the mice were able to consume their daily water allowance inside the MRI with satisfactory sniff signals obtained, we started the odor discrimination phase, in which two neutral odorants were used for a go/no-go paradigm.

During lick training, mice were acclimated to head fixation, the MRI environment, and drinking from the water port was reinforced. In these sessions, mice were head fixed and received water rewards for licking the water port with gradually increased trial durations (and as a result the inter-reward interval). The first session was conducted inside the MRI room, but outside the scanner bore to allow adjustment of the water port location with trial duration of 2.5 s. The next phase was done inside the bore with inter-trial interval of 3–7 s, and odor port positioned in front of the mice. The final phase included inter-trial interval of 5–10 s with full sniff recording. When mice consumed their daily water portion inside the scanner (estimated by number of rewards and change in weight), odor discrimination was started. The first day of odor discrimination included 10–15 min of lick training during scanner calibration, to allow acquisition of fMRI data of the first odor presentation. The next sessions started with one to two 6 minutes blocks of odor discrimination (25 trials per odor) before data were acquired. Importantly, Optseq software was used to design the rapid-presentation event-related experiment (Dale, 1999); inter-trial interval ranged between 5 and 12.5 s. Several sessions (0.6 ± 0.84, range 0–2) were excluded from the fMRI analysis due to insufficient data (less than three blocks with fMRI scans) or drastic decreases in performance (Supplementary Fig. S1), but were still included in the behavioral data presented in the Results Section. Additionally, for some mice, there were few days (overall 0.5 ± 0.71, range 0–2) in which we started the experiment, but mice did not cooperate or perform. Such cases were defined as invalid sessions and removed. Overall, about 85% (64/75) of scanning sessions were included in the fMRI analysis. The final dataset included 10 mice with 6.5 ± 1.08 sessions (range 5–8) and a total 34.6 ± 6.1 blocks per mouse (range 26–41, equal to 207.6 ± 36.6 min of data).

### 2.4 Odorant delivery and high-resolution behavioral monitoring

We designed an MRI-compatible cradle that supports olfactory-guided behavioral experiments. The platform includes a head-fixation apparatus (Bergmann et al., 2016), an odorant-delivery system (olfactometer) for fast and precise odorant presentation, a non-invasive sniff sensing sensor, and a lick detector for behavioral monitoring, and a microliter-resolution system for delivery of water reward. Several changes were made to adapt the classic setup for olfactory-guided behavior in head-fixed mice (Smear et al., 2011) to the MRI environment: (1) The olfactometer was positioned in the preparation room, with its output connected to a final solenoid valve located a short distance outside the scanner bore (Supplementary Fig. S2). The elongation of the path from the final solenoid valve to the mouse odor port, compared with the classic design, only moderately prolonged odor onset latency, with concentration reaching steady state ~100 ms after final valve opening. (2) Sniff recording was performed non-invasively using a novel design of the odor port, enabling both delivery of odorants and simultaneously sampling of pressure changes from sniffing. The sniff port was connected to a pressure sensor with a ~15 cm long, capillary tubing, and optimally positioned to reduce signal artifacts from the scanner. Remaining gradients-induced artifacts in the sniff signal were eliminated using a lowpass filter. (3) Lick detection was based on a commercial MRI-compatible respiration monitor which was modified to detect pressure changes elicited by the animal licking a water tube. (4) A water reservoir was positioned outside the scanner room, with water delivery to the animal gated by a solenoid valve which was calibrated to deliver consistent volumes across sessions (~2.5 µl per drop). Critically, the addition of these components to the animal cradle in the MRI environment did not affect the MRI signal, enabling good signal-to-noise ratio (SNR) and image homogeneity (Supplementary Fig. S3).

For odor stimulus delivery, we used an eight-channel, custom-built, air dilution olfactometer, the design of which has been previously described in detail (Arneodo et al., 2018; Shusterman et al., 2011). Briefly, approximately 1 s prior to odorant delivery onset to the animal, a stream of nitrogen was switched from a blank channel and diverted through one of the odorant vials. The nitrogen carrier pushed the now odorized stream of 100 ml/min, exiting the vial, and quickly merging into the main clean air stream, which had a flow rate of 892 ml/min and resulting in ~10-fold air dilution. The total odorized stream of 992 ml/min was homogenized in a long thin capillary (~5 m) before reaching a final valve, which controlled output to the odor port. Any period of time that was not a part of the stimulus period, such as during the inter-trial interval, a steady stream of clean air with the same rate (992 ml/min) flowed to the odor port continuously. Simultaneously, the flow from the olfactometer was directed to an exhaust during any non-stimulus time. All gas flows were controlled by mass flow controllers (Alicat MC series, MC-1SLPM-D/5M/5IN and MC-100SCCM-D/5M/5IN) with 0.5% accuracy. During stimulus delivery, the final valve (four-way Teflon valve, NResearch, SH360T042) switched the olfactometer odorant stream to the odor port in front of the animal and diverted the clean airflow to an exhaust. Thus, the animal always experienced a constant flow of air, with or without odorant, and as a result of matching the flows, the transition from odorless to odorized air was quick and seamless.

Temporal odorant concentration profiles were checked by a mini photoionization detector (PID, Aurora Scientific, model 200B). The concentration reached a steady state ~100 ms (odor-dependent) after final valve opening. To minimize pressure shocks and provide temporally precise, steplike, reproducible, and fast odorant delivery, we matched the flow impedances of the odor port and exhaust lines, as well as the flow rates from the olfactometer and clean air lines.

At the end of the odorant delivery (1 s duration), the final valve was deactivated, and the clean air line switched back to the odor port. Shortly after, the nitrogen flow in the olfactometer was diverted from the odorant vial to a blank channel, with no odorant. Inter-odor delivery interval was 4–11.5 s, during which clean air was flowing, at the same rate, through all olfactometer flow paths, made from Teflon and Teflon tubing. All odorants (purchased from Sigma-Aldrich) were diluted in mineral oil (dilution 1:100) and stored in liquid phase in dark vials. Each vial contained 2 ml of mineral oil with diluted odorant and 48 ml of headspace.

Non-invasive sniff recording was acquired from the odor port itself, which was connected to a ± 0.5 PSI pressure sensor (Honeywell, 24PCEFJ6G) using a ~15 cm tube. The placement and length of the connection were optimized in order to reduce gradients-induced artifacts. The sensor was connected to a custom-made amplifier and filter, and sampled using an Arduino Leonardo microcontroller (https://www.arduino.cc/). In addition, Arduino Leonardo gated final valve opening, controlled water delivery using a second solenoid valve (Asco, SCH284B007), and sampled the pressure signal from the lick detector. This detector was based on the respiration sensor and amplifier of 1025 MR-compatible monitoring systems (Small Animal Instruments, Inc.) equipped with a signal breakout module. Recording of all signals, online lick detection, and water delivery was done using in-house scripts in MATLAB.

### 2.5 Image acquisition

MRI scans were performed at 9.4 Tesla MRI (Bruker BioSpin, Ettlingen, Germany) using a quadrature 86 mm transmit-only coil (Bruker BioSpin) and a 20 mm loop receive-only coil (Bruker BioSpin). Mice were briefly anesthetized (5% isoflurane) and mounted on the cradle. Odor and water ports were adjusted until proper sniff recording and access to water reward were achieved. Each daily session included acquisition of one low-resolution rapid acquisition process with a relaxation enhancement (RARE) T2-weighted structural volume (50 coronal slices, TR/TE 2300/8.5 ms, RARE factor = 4, flip angle = 180°, 200 × 200 × 300 μm^3^, field of view of 19.2 × 19.2 mm^2^, matrix size of 96 × 96) and multiple 6 min spin-echo echo-planar imaging blocks measuring BOLD (TR/TE 2500/13.022 ms, flip angle = 90°, 50 coronal slices, 200 × 200 × 300 μm^3^, field of view of 14.4 × 9.6 mm^2^, matrix size of 72 × 48; the imaged volume was framed with 4 saturation slices to avoid wraparound artifacts). During scanner calibrations and acquisition of anatomical images, mice started to perform the odor discrimination task (25–50 trails per odor), except for the first session in which lick training protocol was used in order to image the first exposure to odors.

### 2.6 Data preprocessing and analysis

Standard fMRI preprocessing was performed as previously described (Desai et al., 2011). Briefly, the procedure included removal of the first two volumes for T1-equilibration effects, compensation of slice-dependent time shifts (SPM), rigid body correction for head motion and linear registration (FSL FLIRT) to the Allen Mouse Brain Common Coordinate Framework version 3 (CCFv3, Kuan et al., 2015; Lein et al., 2007), and a spatial smoothing with a Gaussian kernel of 600 μm. Importantly, linear registration was semi-automatic with manual correction for each session to validate the alignment of the olfactory bulb, striatum, hippocampus, and frontoparietal cortices.

### 2.7 Modeling the HRF

Since SPM canonical HRF is not optimized to capture brain responses in other species (Boch et al., 2021; Lambers et al., 2020), and prior studies in mice have modeled the HRF during passive sensory stimuli in anesthetized (Kahn et al., 2011) and awake (Chen et al., 2020) states, we sought to characterize the HRF in behaving mice. We used the Allen Mouse Brain Atlas to extract hemodynamic responses in pre-defined regions that are known to be involved in the task (Supplementary Fig. S4). Out of the four tested regions, the ventral striatum (VS) showed the most robust response, which was used to model the HRF by scanning different parameters. This region was also selected to align with our goal of investigating the role of subcortical memory systems in learning and goal-directed behavior. We first extracted VS response to Hit trials from all scans of each mouse and then averaged the HRF across animals. This averaged response was used for testing the different parameters to estimate a tailored behaving mouse HRF. We scanned and optimized different parameters used by spm_hrf.m function, namely the delay of the response (p1 = 3 s, range scanned = 1:0.1:6), the delay of the undershoot (p2 = 4.8 s, range scanned = 1:0.1:16), the dispersion of the response (p3 = 1.4, range scanned = 0.5:0.1:1.5), the dispersion of the undershoot (p4 = 0.6, range scanned = 0.5:0.1:1.5), the relation of the response to the undershoot (p5 = 2, range scanned = 2:1:20), the onset (p6 = 0 s, not scanned) and length of the kernel (p7 = 30 s, not scanned). The different HRFs were convolved with a stick function to generate the main response regressor at fMRI temporal resolution, the described parameters yielded minimal difference between the observed HRF and modeled response as estimated using root-mean-square error, and thus used in further voxel-wise GLM analysis. This model successfully captured distributed brain responses, with the exception of the main olfactory bulb (MOB). This finding is consistent with temporal discrepancies observed in region of interest (ROI) analysis. To account for these differences, we scanned a range of temporal shifts (p6) and computed RMSE between the shifted HRF and the average MOB response. A 2-s shift yielded the lowest error, and a GLM based on this adjusted HRF successfully captured responses in the MOB (Supplementary Figs. S4 and S5). Technical limitations of HRF estimation are detailed in the Discussion section.

### 2.8 Motion

A possible confound in task-based fMRI is head motion, which is known to be associated with image artifacts. This confound is especially relevant to our task design which involves active licking behavior in the Hit and False Alarm (FA) conditions. To address this issue, we quantified head motion in different trials and its effects on task-evoked responses (Supplementary Fig. S6). While we found increased head motion in Hit and FA trials compared with correct rejection (CR) trials, the absolute motion values were relatively low, and only small fraction of trials were contaminated by motion higher than the size of a single voxel. Critically, comparing trials with high or low head motion values, we found close agreement between the results at the level of both ROI and voxel-wise analyses. Thus, while head motion was overall low (average frame displacement per mouse: 37.3 ± 7.53 μm, mean ± SD), Hit, FA, and CR trials with head motion higher than diameter of one voxel (average frames per mouse: 2.89 ± 1.37, mean ± SD) were moved to the fourth condition and not included in the analysis. A possible explanation for the resiliency to motion artifacts is the use of nuisance regressors based on the CSF and global signals. This preprocessing step, which is uncommon in task-based fMRI increased the specificity of statistical maps (cf. Supplementary Fig. S7). Yet, it is likely that it also reduces the observed responses, especially in somatomotor regions. Given the differences between rodent and human task-based fMRI design, namely activation of facial muscles for lick responses, and frequency of heart and respiratory rates, we chose a very conservative preprocessing. While we still observed significant task-evoked responses, future studies may use more lenient noise-reduction techniques to maximize the observed signals.

## 3 Statistics and Reproducibility

### 3.1 Whole-brain analysis

fMRI analysis was conducted using SPM (Wellcome Department of Cognitive Neurology, London, UK). Hit, CR, and FA trials were defined as separate conditions. Misses, random licks, and manual rewards from early trials were defined together as a fourth condition, which also included CR trials with adjacent licks outside the response window. We decided to exclude Miss trials as a standalone regressor because after task acquisition these trials were infrequent. Preliminary ROI analyses of Miss trials (data not shown) did not reveal consistent activation across animals. In addition to these four conditions, three translation and three rotation motion parameters were defined as nuisance regressors, as well as ventricular and whole-brain signals and their derivatives (Supplementary Fig. S7). A GLM analysis was used to characterize whole-brain responses for different task conditions. For each mouse, multiple blocks and sessions were concatenated to extract a single beta estimates map per contrast (34.6 ± 6.1 blocks, mean ± SD). Then, these maps were submitted to a second-level group analysis using SPM; the resulting *t*-maps were corrected for multiple comparisons using the false-discovery rate method (Benjamini & Hochberg, 1995). Further ROI analyses were also conducted Using MarsBaR.

### 3.2 ROI analysis

ROI analysis was performed using MarsBaR toolbox (Brett et al., 2002) to extract finite impulse responses. ROIs were defined using parcels from the Allen Atlas or clusters from the statistical maps. For the characterization of responses within the mouse striatum, ROIs were manually drawn. Comparisons of response amplitudes were done using the peak percent signal change at the third time point using two-tailed paired and unpaired Student’s *t*-tests. For the comparisons between slow and fast reaction times, the Hit trials were sorted by their reaction times, the fast condition included the fastest third of trials within each block, while the slow condition included the slowest third. The comparison between naïve and expert stages was done by restricting the analysis to the first and last 10 blocks, respectively, and comparing percent signal changes in a paired manner; the number of fMRI blocks between these states varied between mice (14.6 ± 6.096 blocks, mean ± SD).

## 4 Methodological Limitations

While the experimental setup presented in this paper enabled characterization of longitudinal brain dynamics in behaving mice from naïve to expert stages, the current study has several limitations.

The presented platform supports high-resolution behavioral monitoring inside the MRI scanner. While basic analyses of behavioral measures were conducted including analysis of sniff and lick signals and learning-related dynamics within the striatum, future studies will dissect different behavioral parameters and individual learning patterns to better characterize the evolution of task proficiency and its effects on distributed brain response. The described analyses revealed differential responses of the mouse hippocampal and striatal memory systems to Hit and FA, with fine grained response pattern within adjacent striatal subregions. However, given the small sample size, further experiments should examine the reproducibility of these findings.

Previous reports demonstrated the feasibility of odor-based task-based fMRI in behaving mice (Fonseca et al., 2022; Z. Han et al., 2019; Winkelmeier et al., 2022). However, in these studies, mice were trained outside the scanner with no fMRI data collected during task acquisition. Several technical advancements distinguish the presented setup, allowing for longitudinal imaging over learning, including shorter odor onset latency that supports shorter inter-trial interval (5 s compared with 10 s in previous reports). Second, a better control of odor presentation was achieved using mass flow controllers and active evacuation of odorized air, assuring that mice respond to odor identity and not to pressure changes associated with odor presentation (Z. Han et al., 2019; Winkelmeier et al., 2022). These components that are lacking in other setups may reduce the stability of behavioral performance, as seen in the work of Han et al. in which the performance levels inside the MRI were lower than in out-of-scanner experiments (Z. Han et al., 2018, 2019). We argue that such stability is a pre-requisite for in-scanner task acquisition, which is necessary for characterization of learning processes.

Another important advancement of the presented setup is the incorporation of high-resolution behavioral monitoring into an MRI-compatible behavioral platform. The ability to record sniffing enables examination of active sensing behaviors which are known to affect stimulus perception in mice (Smear et al., 2011) and humans (Mainland & Sobel, 2006). Importantly, respiration itself has shown to entrain brain rhythms including hippocampal oscillations (Tort et al., 2018). Sniff recording allows for a more accurate estimation of stimulus onset and, consequently, a better estimate of reaction time, which has previously been shown to explain trial-by-trial variability in fMRI (Yarkoni et al., 2009). Here we showed that learning affects both sniffing and reaction time, and that the latter also explains variability in brain responses within and between mice. Future studies will further characterize the changes in these measures over learning and their neural signatures.

## 5 Results

### 5.1 Odor discrimination learning

**Fig. 1.**
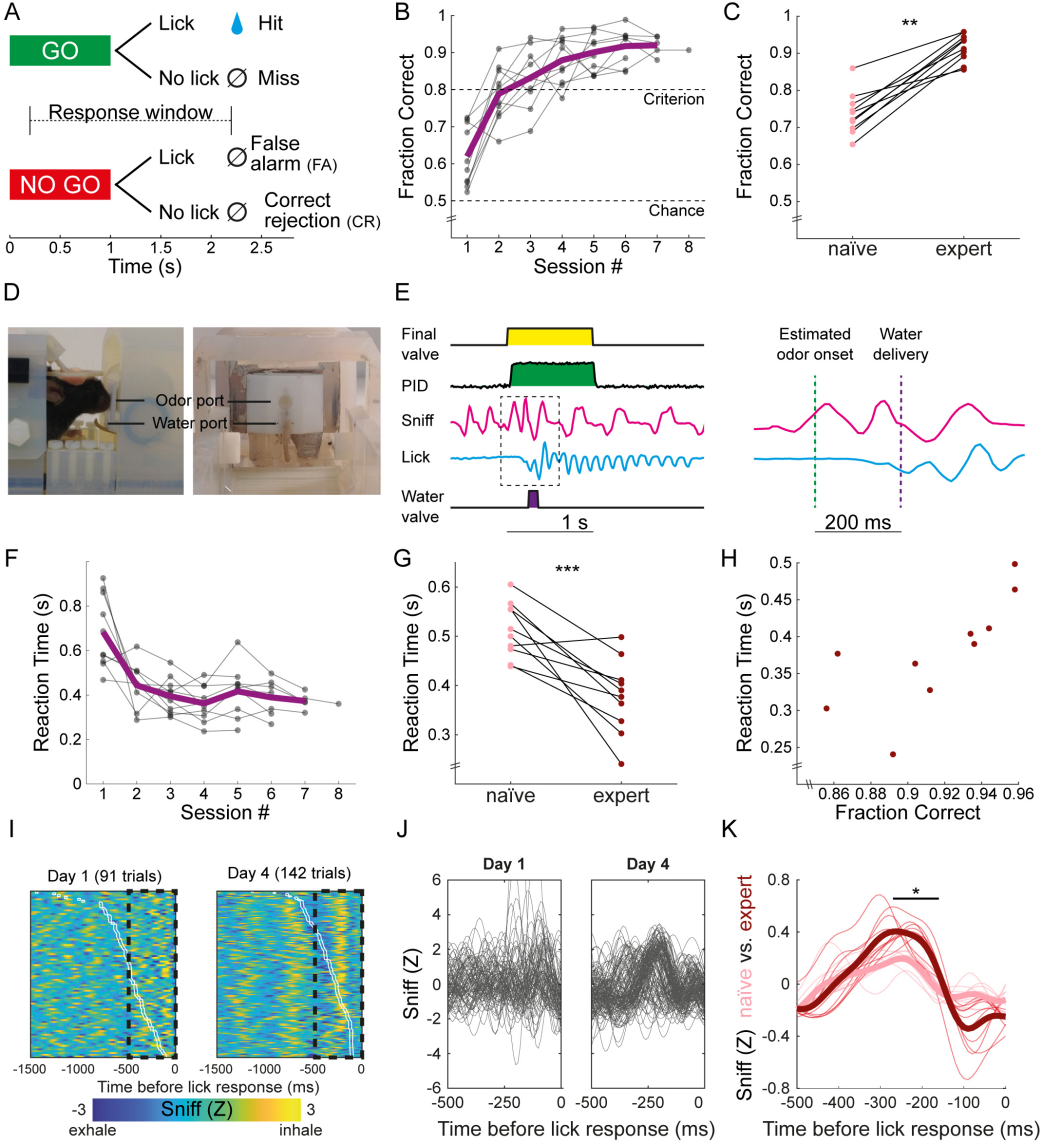
Odor discrimination learning: task-based mouse fMRI with high-resolution behavioral monitoring. (A) Task structure. Odors were presented for 1 s; response window started 200 ms after final valve opening and lasted 2 s. (B) Learning curves demonstrate discrimination between odors in all 10 mice. (C) A comparison between task performance in naïve and expert stages (average of first and last 10 blocks, respectively). (D) Experimental setup. A head-ﬁxed mouse is shown in the cradle with a 20 mm receive-only loop coil located above the head (*left*); front view of the odor and water ports allow non-invasive sniff recording and delivery of water reward, respectively (*right*). (E) High-resolution behavioral monitoring integrates multiple signals related to odorant presentation (final valve opening, odorant pulse profile as measured with a photoionization detector [PID]), mouse sniffing, lick detection, and water delivery (*left*). A zoom on the sniffing and licking signals following odor onset and water delivery (*right*). (F) Individual mean reaction times in Hit trials demonstrate faster reaction times to odors concomitant with the animal reaching criterion. (G) A comparison between reaction times in naïve and expert stages. (H) A comparison between performance and reaction time in expert mice demonstrates that task accuracy is associated with slower responses. (I) Sniffing behavior before lick response in each Hit trial in a single session is shown for a representative mouse at day 1 (*left*) and day 4 (*right*). The white outline (overlaid on the sniff responses) demonstrates a decrease in reaction time (interval between odor onset and lick response at time zero) over learning. (J) A zoom on sniffing behavior that preceded licking in individual trials (dashed rectangle in panel I) is shown for day 1 (*left*) and day 4 (*right*), demonstrating association between olfactory stimulus and lick response over learning. (K) A comparison between average sniff signals in naïve and expert stages confirms shaping of the sniff signal. *P < 0.05, **P < 0.01, ***P < 0.001, paired Student’s *t*-test.

Ten mice were included in a go/no-go odor discrimination task (Fig. 1A; Supplementary Video S1; Supplementary Fig. S1). After pre-training mice to lick a water port to receive rewards, scanning commenced. Half of the mice received a water reward when responding with a lick for pinene (“go”) but not for ethyl-acetate (“no-go”), and half were trained to lick for ethyl-acetate and avoid licking for pinene. All mice reached performance criterion (Fig. 1B), overall taking between 150 and 875 trials before the first daily session that was above criterion (368.8 ± 216.7 trials, mean ± SD; each daily session consisted of several blocks of 25 “go” and 25 “no-go” trials). Examination of individual learning curves (Supplementary Fig. S1) revealed inter-subject variability with some mice learning the task over a single session. Comparing between naïve and expert stages of each mouse (the first and last 10 six-minute fMRI scanning blocks, spanning roughly the first and last 2–3 days, respectively), we found that all mice improved their performance during learning (Fig. 1C, naïve: 0.737 ± 0.057; mean ± SD; expert: 0.916 ± 0.037; two-tailed paired Student’s *t*-test: *t*_(9)_ = 8.96, P < 0.001, Cohen’s d = 2.834). Moreover, stable performance in later stages was not associated with the improvement in performance during early stages of acquisition (Spearman’s ρ = 0.17, P = 0.683), suggesting that while learning rate was variable across mice, consistent stable performance was achieved after mice reached criterion and rate of learning did not impact it.

Reaction time and adjustments of odor detection were evaluated using the recorded licking and sniffing responses (Fig. 1D, E). Reaction time was defined as the time between odor presentation and the first lick of the water port. We found that task proficiency is associated with a faster lick response. The impact of experience was evaluated by submitting daily average reaction times of the first five sessions (Fig. 1F) to a repeated-measures ANOVA (corrected for sphericity violation using the Huynh–Feldt method) with time as within-subject factor. We observed a significant effect of Time (F_(4, 36)_ = 15.4, P < 0.001, ε_H-F_ = 0.498, η^2^ = 0.548), indicating that reaction time decreased with learning. Direct comparison between naïve and expert stages (Fig. 1G) supported this observation (two-tailed paired Student’s *t*-test: *t*_(9)_ = 4.63, P = 0.001, Cohen’s d = 1.47). Interestingly, reaction time and task performance were not correlated in the naïve stage (Spearman’s ρ = -0.38, P = 0.279) but were in the expert stage (Spearman’s ρ = 0.875, P < 0.001), revealing a speed–accuracy tradeoff only when animals became proficient (Fig. 1H).

Examining representative sniff patterns from early and late stages of learning, we found that when the animal achieves task proficiency, an association between the odor stimulus and the sniff response is formed (Fig. 1I), resulting in synchronized sniff cycle before licking (Fig. 1J). Comparing average sniff signals between naïve and expert stages, we confirmed this observation, demonstrating increased average sniff amplitude before licking in expert mice (Fig. 1K). Collectively, these results suggest that mice consistently achieved above criterion performance allowing for proper interpretation of brain responses measured with fMRI.

### 5.2 Whole-brain analysis of instrumental learning using event-related fMRI in mice

**Fig. 2.**
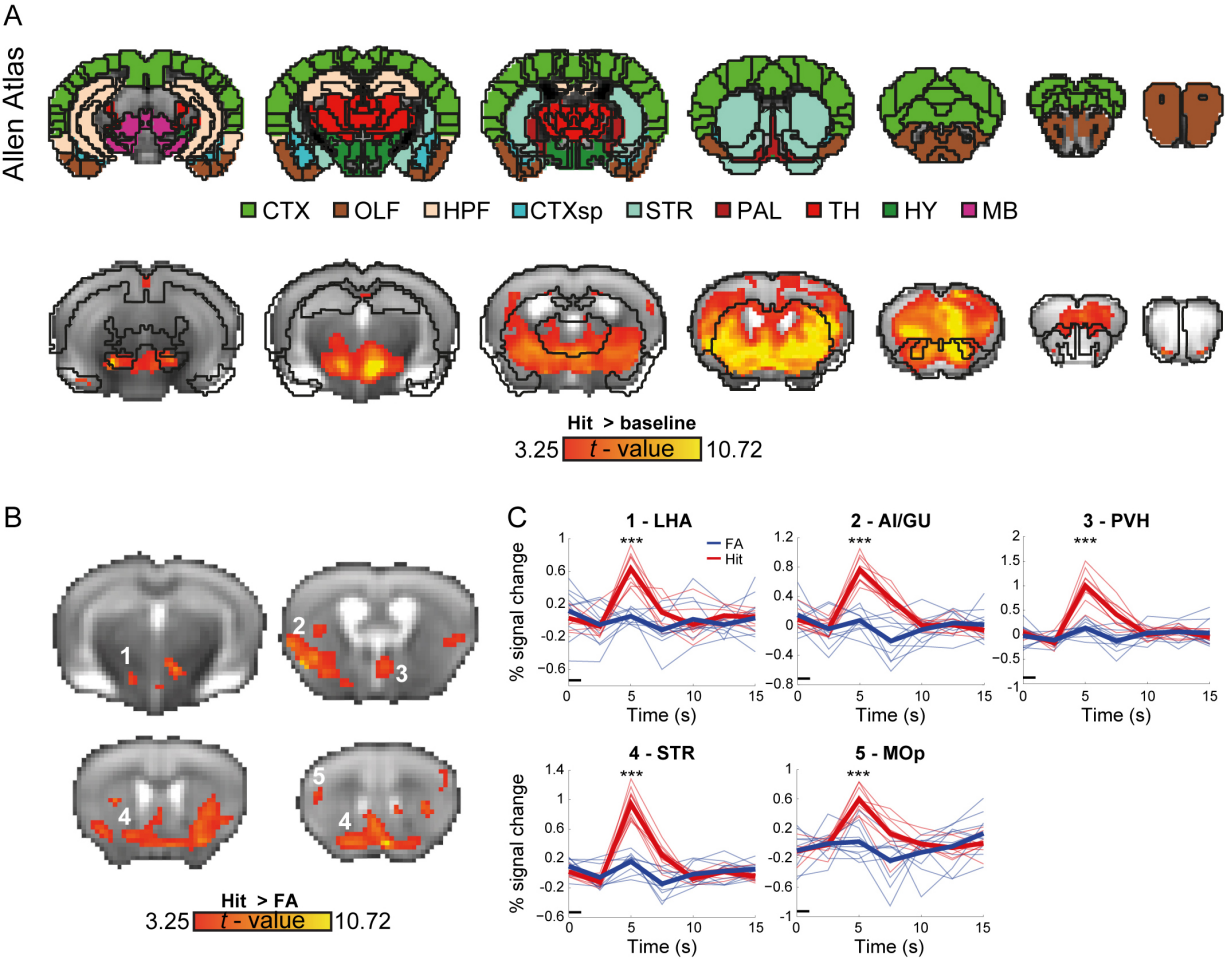
Whole-brain analysis of task-evoked responses to reward. (A) Labels from the Allen Mouse Brain Atlas (*top*) were overlaid on group statistical parametric maps (n = 10 mice). The analysis uncovered activations associated with correct “go” trials (Hit) in somatomotor and high-order cortices, as well as subcortical structures, demonstrating prominent involvement of the striatum; maps were corrected for multiple comparisons using the false-discovery rate method (P < 0.05, voxel extent of 5) and presented over a group average fMRI image (200 × 200 × 300 µm^3^ voxel resolution). CTX—cerebral cortex; CTXsp—cortical subplate; HPF—hippocampal formation; HY—hypothalamus; MB—midbrain; OLF—olfactory areas; PAL—pallidum; STR—striatum; TH—thalamus. (B) A statistical parametric map of Hit greater than false alarm “no-go” trials (Hit > FA); the maps are presented over the group average fMRI image) and annotated based on labels from the Allen Mouse Brain Atlas; P < 0.05, corrected for multiple comparisons using false-discovery rate correction, voxel extent of 5. (C) ROI analysis of Hit (*red*) and FA (*blue*) responses in the clusters identified using the GLM. The black line depicts the onset of the odor stimulus relative to the hemodynamic response ***P < 0.001, corrected for multiple comparisons using the Bonferroni method.

First, we sought to identify brain responses to perceptual and cognitive processes implicated in the go/no-go instrumental learning task. We compared correct go responses (Hit) with baseline periods (inter-trial intervals), which captures responses to odor perception (sniff), motor response (lick), reward (water delivery), and ingestion (water consumption). We modeled the HRF using a GLM that was objectively derived from the data. The analysis revealed distributed responses in somatomotor cortices, high-order association cortices, and subcortical structures, demonstrating prominent involvement of the striatum (Fig. 2A; Supplementary Table S1). While the GLM analysis did not capture responses in olfactory regions, they could be detected using the modified version of the HRF (Supplementary Figs. S4 and S5).

Next, we compared responses with Hit and FA (a no-go trial that the animal responded to with a lick) conditions, which share perceptual, decision making, and motor control features, and differ primarily in aspects of reward processing and ingestion. In both conditions, mice receive an olfactory stimulus and lick the water port with an expectation for water, thus potentially perceiving in both cases the odor as a “go” signal (or for FA failing to perceive it as a “no-go” signal), but rewards are given only in Hit and not in FA trials. Contrasting these conditions (“Hit > FA”), we identified several significant regions (Fig. 2B; Supplementary Table S2), demonstrating preferential responses to Hit in primary motor (MOp) and insular/gustatory (AI/GU) cortices as well as in the sub-cortical structures, lateral hypothalamic area (LHA), and striatum (STR).

Since the HRF in our GLM model was defined using responses in Hit trials, we wanted to validate that the results of the whole-brain analysis do not stem from differences in the HRF between these conditions. Thus, to better characterize the responses of the identified clusters to Hit and FA trials, we conducted an ROI analysis (Fig. 2C), which confirmed the GLM results by demonstrating significant differences in all regions (Supplementary Table S3). The observation that the motor and insular/gustatory cortices, the hypothalamus, and striatum are selectively engaged in Hit, but not FA, trials may suggest that they subserve neural processes related to the consumption of water reward and drinking behavior. These findings are consistent with previous reports on conjunction of reward and ingestive behaviors in the hypothalamus (Bernardis & Bellinger, 1996; Stuber & Wise, 2016), and in line with the motor and gustatory aspects of drinking.

### 5.3 Striatal subregions differentially contribute to separable task performance aspects

**Fig. 3.**
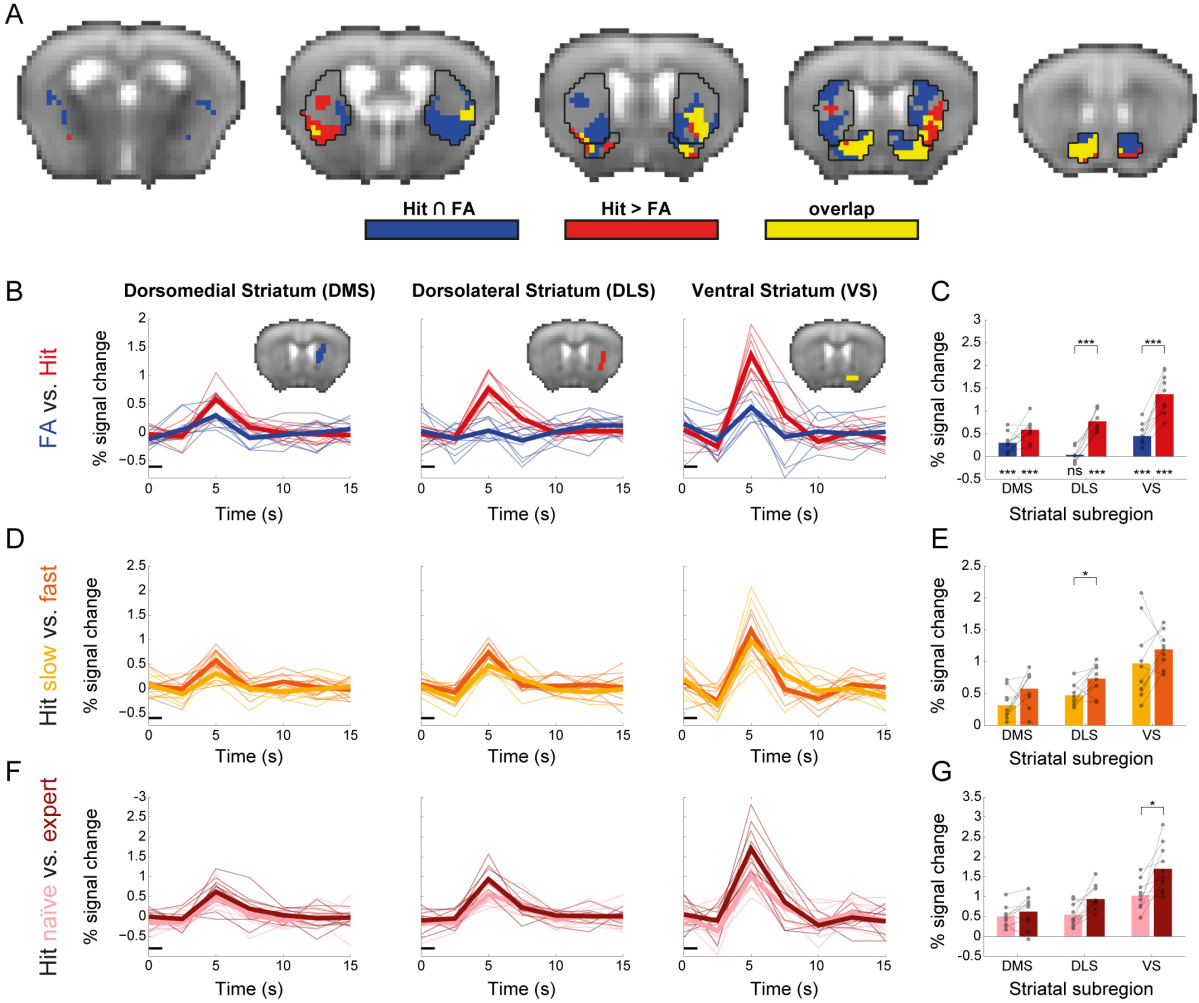
Striatal contributions to task performance and veridical memory responses. (A) Spatial organization of overlapping Hit over baseline and FA over baseline responses (*blue*), preferential Hit responses (*red*), and overlap between them (*yellow*) in the mouse striatum; maps were thresholded using a statistical threshold of P < 0.05, corrected for multiple comparisons using false-discovery rate correction, voxel extent of 5. (B) ROI analysis of Hit and FA in the dorsomedial (DMS), dorsolateral (DLS), and ventral (VS) striatal regions. The black line depicts the onset of the odor stimulus relative to the hemodynamic response. (C) Comparison of response amplitude (peak signal change) for Hit and FA in the three striatal ROIs reveals that while all regions respond to Hit, DMS responds similarly to FA, DLS does not respond to FA and VS responds to FA, but its response to Hit is stronger. *P < 0.05, ***P < 0.001. (D) ROI analysis of striatal activation in Hit with slow (*yellow*) and fast (*orange*) reaction times. (E) Comparison of response amplitudes between Hit with slow and fast reaction times reveals significant differences in DLS only. *P < 0.05. (F) ROI analysis of striatal activation to Hit in naïve (*pink*) and expert (*dark red*) stages of learning. (G) Comparison of response amplitudes between Hit in naïve and expert stages reveals significant differences in VS only; *P < 0.05.

The striatum is known to participate in many cognitive processes involved in the odor discrimination task such as motor control, decision making, and reward processing (Cox & Witten, 2019). Comparing the “Hit > baseline” and “Hit > FA” maps, we observed spatial differences in responses within the striatum. While the comparison with baseline yielded spatially distributed striatal responses, comparison with FA resulted in more localized differences as a significant preferential response to Hit was identified in a relatively small number of striatal voxels. Therefore, we sought to quantify the contribution of different striatal sub-regions in the odor discrimination task. To do so, we characterized the spatial overlap between two maps: the “Hit > FA” contrast which represents responses associated with reward processing and the conjunction map between “Hit > baseline” and “FA > baseline”, which represents voxels that are significant in both contrasts, and thus are more associated with goal-directed behavior (Z. Han et al., 2019). The analysis revealed separation between the maps in the dorsal striatum (Fig. 3A). The dorsomedial striatum (DMS) demonstrated majority of voxels from the conjunction of “Hit > baseline” and “FA > baseline”, with no evidence for a preferential response to either condition, while dorsolateral striatum (DLS) exhibited a preferential response to Hit with no evidence for conjunction. In contrast, ventral striatum (VS) exhibited both conjunction and a preferential response to Hit.

To better characterize the differences in striatal responses to Hit and FA trials, we conducted an ROI analysis (Fig. 3B, C), which was corrected for multiple comparisons using the Bonferroni method adjusted alpha level of 0.0056 per test (0.05/9). The results confirmed our observations as DMS responded similarly to both conditions (Hit: 0.584% ± 0.237, mean ± SD, two-tailed one-sample Student’s *t*-test: *t*_(9)_ = 7.79, P < 0.001; FA: 0.299% ± 0.206, *t*_(9)_ = 4.59, P = 0.013) with Hits numerically higher than FA but not reliable when correcting for multiple comparisons (Hit > FA: two-tailed paired Student’s *t*-test: *t*_(9)_ = 2.9, P = 0.018, Cohen’s d = 0.31). VS responded to both conditions, but the response to Hit was significantly higher (Hit: 1.366% ± 0.417, *t*_(9)_ = 10.35, P < 0.001; FA: 0.446% ± 0.236, *t*_(9)_ = 5.98, P < 0.001, Hit > FA: *t*_(9)_ = 7.37, P < 0.001, Cohen’s d = 1), while the DLS responded only to Hit trials (Hit: 0.769% ± 0.237, *t*_(9)_ = 10.28, P < 0.001; FA: 0.033% ± 0.176, *t*_(9)_ = 0.6, P = 0.561, Hit>FA: *t*_(9)_ = 7.47, P < 0.001, Cohen’s d = 1.17). These results confirm the findings from the volume voxel-wise analysis, capturing differential responses in adjacent striatal subregions.

The previous analysis uncovered a variety of responses within the striatum during the task, which can putatively link to different associations between stimulus, response, and outcome. To better dissociate between these processes, we sought to examine whether striatal sub-regions are differentially sensitive to motor variables, namely reaction time. Therefore, we compared striatal responses in Hit trials with slow and fast reaction times, a contrast that isolates decision making from motor aspects, as reward size is constant (Fig. 3D, E). Since animals learn relatively quickly in this task, FA trials are relatively infrequent, thus could not be split by reaction time, so this analysis was restricted to Hit trials; again, adjusting for multiple comparisons using Bonferroni-adjusted alpha levels of 0.0167 per test (0.05/3). We observed preferential responses to trials with fast reaction times in the DLS (slow RT: 0.471% ± 0.174, fast RT: 0.733% ± 0.233; two-tailed paired Student’s *t-*test: *t*_(9)_ = 3.014, P = 0.015, Cohen’s d = 0.95), a trend toward significance in DMS (slow RT: 0.315% ± 0.223, fast RT: 0.575% ± 0.273; *t*_(9)_ = 2.266, P = 0.05, Cohen’s d = 0.72) and RT-independent response in the VS (slow RT: 0.973% ± 0.591, fast RT: 1.193% ± 0.261; *t*_(9)_ = 1.288, P = 0.23, Cohen’s d = 0.41), suggesting that the dorsal, but not ventral, striatum is affected by decision making and motor aspects of the task, and consistent with the role of DLS in skilled movement and habitual actions.

Finally, another feature that can further delineate goal-directed behavior and reward processes within the striatum is the dynamics of different sub-regions during learning. Comparing striatal Hit responses in naïve and expert stages (Fig. 3F), we found differential effects of task proficiency within the striatum (Fig. 3G), with VS showing significantly stronger responses in expert mice (naïve: 1.022% ± 0.366, expert: 1.701% ± 0.599; *t*_(9)_ = 3.337, P = 0.009, Cohen’s d = 1.06), DLS demonstrating marginally significant preferential responses in experts (naïve: 0.546% ± 0.279; expert: 0.939% ± 0.328; two-tailed paired Student’s *t-*test: *t*_(9)_ = 2.906, P = 0.017, Cohen’s d = 0.92), and DMS exhibiting stable responses during learning (naïve: 0.504% ± 0.255; expert: 0.625% ± 0.395; *t*_(9)_ = 1.414, P = 0.191, Cohen’s d = 0.45), suggesting that the latter is less affected by association related to the olfactory stimulus as these associations developed during learning (statistical thresholds were adjusted for multiple comparisons using Bonferroni-adjusted alpha levels of 0.0167 per test). Whole-brain analysis of learning effects on Hit responses did not meet conservative thresholds for correction for multiple comparisons. However, the results of the ROI analysis were captured at lenient statistical thresholds, in addition to other cortical and subcortical clusters (Supplementary Fig. S8). Collectively, the analysis of striatal contribution to the odor discrimination task recapitulated the known functional sub-division of the striatum.

### 5.4 Hippocampal responses underlie false memory

**Fig. 4.**
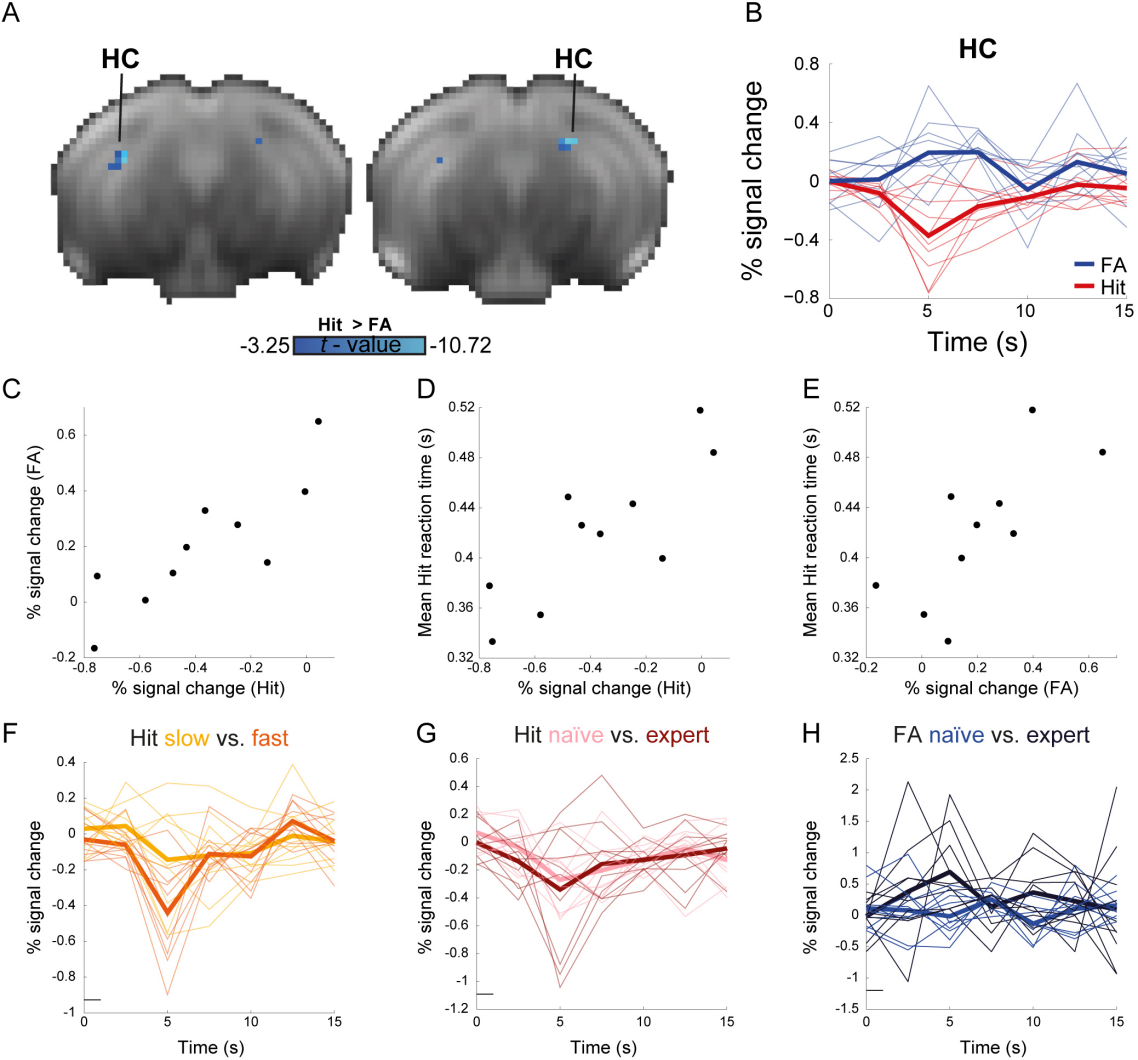
Hippocampal contributions to task performance. (A) A negative statistical parametric map of HIT > FA contrast reveals a significant difference in dorsal hippocampus (HC); P < 0.05, corrected for multiple comparisons using false-discovery rate correction, voxel extent of 5. (B) ROI analysis of hippocampal responses to Hit and FA. (C) Relationships between HC responses to Hit and FA. (D) Relationships between individual variation in mean reaction time and HC responses to Hit. (E) Relationships between individual variation in mean reaction time and HC responses in FA. (F) ROI analysis of hippocampal negative response to Hit with slow and fast reaction times, demonstrating a marginally significant difference. (G) ROI analysis of hippocampal negative response to Hit in naïve and expert stages. (H) ROI analysis of hippocampal activation to FA in naïve and expert stages demonstrating shaping of HC response with learning.

Next, we sought to identify regions contributing to false memory responses. Examining the negative statistical map of the “Hit > FA” contrast, we identified bilateral clusters in the hippocampus and parahippocampal region/posterior piriform cortex which exhibited a preferential response to FA (Fig. 4A); due to the proximity of the latter to the ear canal and its potentially associated artifacts, we focused on the hippocampus. ROI analysis of the clusters in the hippocampus revealed a differential response, demonstrating negative response in Hit trials when compared with FA trials (Fig. 4B). This response was not limited to the identified clusters and was replicated in a whole-hippocampal ROI analysis with no localization to a specific subfield (Supplementary Fig. S9). Critically, examining the relationships between Hit and FA responses, we found that they are highly correlated (Fig. 4C; Spearman’s ρ = 0.903, P < 0.001), suggesting that hippocampal involvement in FA trials is associated with weaker negative response in Hit trials.

To establish the contribution of hippocampal responses to false memory, we sought to examine their behavioral relevance. Comparing between hippocampal responses and average reaction times in Hit trials (Fig. 4D), we found significant correlations in both Hit (Spearman’s ρ = 0.746, P = 0.018) and FA (Spearman’s ρ = 0.782, P = 0.012) trials. To further test the association between hippocampal responses and reaction times within individual mice, we sorted all Hit trials in each run by reaction time and compared the slow and fast tertiles within each mouse (Fig. 4E). The analysis showed that trials with fast reaction times are characterized by stronger hippocampal negative response (-0.439% ± 0.287, mean ± SD) than trials with slow reaction times (-0.144% ± 0.291). A formal comparison revealed marginally significant difference (two-tailed paired Student’s *t*-test: *t*_(9)_ = 2.218, P = 0.054, Cohen’s d = 0.701). Given the limited number of FA trials, we could not characterize a similar pattern in this condition. Importantly, slower reaction times are associated with better performance as shown in Figure 1G.

To better understand the role of this hippocampal response in learning, we compared its involvement between naïve and expert stages (Fig. 4F–H). The results revealed significant interaction between task proficiency and condition (repeated measures ANOVA: F_(1, 9)_ = 35.96, P < 0.001, ε_H-F_ = 1, η^2^ = 0.11). Post hoc analysis showed that Hit trials are characterized by similar negative response in naïve (-0.27% ± 0.221) and expert (-0.34% ± 0.465) stages (two-tailed paired Student’s *t*-test: *t*_(9)_ = 0.458, P = 0.658, Cohen’s d = 0.145). In contrast, FA trials show increased response in the transition from naïve (-0.017% ± 0.35) to expert (0.682% ± 0.632) stage (two-tailed paired Student’s *t*-test: *t*_(9)_ = 3.777, P = 0.009, Cohen’s d = 1.194). These results suggest that hippocampal involvement in FA trials is shaped over learning that the disengagement of the hippocampal memory system allows the striatum to drive correct responses. Moreover, our results indicate that when the animal is proficient in the task, the emergence of strong hippocampal signals drives false memory responses. Collectively, these observations suggest that the hippocampus is involved in learning in a behaviorally relevant manner, albeit negatively impacting performance and competing with striatal responses.

**Fig. 5.**
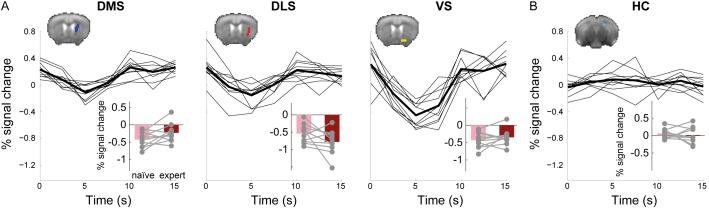
Correct rejection responses are correlated with negative responses in the striatum and no responses in the hippocampus. (A) ROI analysis of correct rejection (CR) responses in the dorsomedial (DMS), dorsolateral (DLS), and ventral (VS) striatal regions demonstrate reliable negative responses. *Insets,* comparison of response amplitude (peak signal change) for naïve and expert stages in the three striatal ROIs reveals that these regions did not differ between naïve and expert stages. (B) CR responses in the dorsal hippocampus (HC) did not reliably differ from baseline nor change between naïve and expert stages (*inset*).

Finally, correct rejection responses were evaluated in both the striatum and dorsal hippocampus. In the striatum, a negative BOLD response profile was evident in all subregions (Fig. 5A). Comparing responses in naïve and expert stages, we did not find any significant effect of learning: DMS (naïve: -0.425% ± 0.218, mean ± SD, expert: -0.232% ± 0.271; *t*_(9)_ = -2.028, P = 0.073, Cohen’s d = 0.641), VS (naïve: -0.543% ± 0.272, expert: -0.78% ± 0.357; *t*_(9)_ = 1.772, P = 0.11, Cohen’s d = 0.561), and DLS (naïve: -0.433% ± 0.23, expert: -0.348% ± 0.228; *t*_(9)_ = -1.042, P = 0.324, Cohen’s d = 0.33). In the dorsal hippocampus, the response profile of correct rejections did not reliably differ from baseline, diverging from both FA that showed a positive response and Hit that showed a negative response (Fig. 5B; cf. Fig 4B). Similar to striatal correct rejection responses, dorsal hippocampal responses did not differ between naïve and expert stages (naïve: 0.065% ± 0.148, expert: 0.016% ± 0.237; *t*_(9)_ = 0.548, P = 0.597, Cohen’s d = 0.173). Altogether, negative responses in the striatum suggest that it plays a role in the no-go correct responses, and the absence of positive or negative responses in the dorsal hippocampus suggests that it is not contributing to response avoidance in the no-go trials.

## 6 Discussion

The memory systems subserving instrumental learning have been extensively studied in humans and rodents. While these experiments contributed significantly to our understanding of memory functions in health and disease, methodological differences in human and rodent brain research have limited translation of findings across species. Here, we used longitudinal task-based fMRI in behaving mice to characterize the contribution of the striatal and hippocampal memory systems to acquisition and performance of a go/no-go odor discrimination task, supporting alignment of scientific questions addressed in humans and mice. The analyses revealed behaviorally relevant competition between these systems with the striatum driving learning of the task and hippocampus contributing to false memory responses.

We first established the brain-wide reward-related responses, revealing consistent engagement of regions comprising components of the reward, olfaction, somatosensory, motor, and water ingestion-related brain regions. Following this analysis, we identified regions subserving veridical (Hit) and false (FA) mnemonic responses. Differences between Hit and FA trials isolate processes associated with reward and feedback, and partly control for motor and olfactory responses, which are shared between the behavioral outputs associated with these conditions. This analysis uncovered several putative clusters in the insular and somatomotor cortices, hypothalamus, striatum, and hippocampus. Yet, the coupling of odor identity and odor valence and the nature of the go/no-go task limited full separation of reward, motor, and olfactory processing for individual mice. While not possible here, future work may disentangle these aspects using incisive behavioral task manipulations. For example, the coupling of odor-identity and odor-valence can be addressed using an odor-detection task in which mice receive rewards for both odorants. Alternatively, the use of a non-deterministic reward outcome or reversal learning paradigms (Izquierdo et al., 2017) can also decouple odor-identity and valence, as well as recruit multiple learning systems (Shohamy et al., 2008). Moreover, a better separation of motor and reward variables can be achieved using a two alternative forced choice task (Chong et al., 2020; Wilson et al., 2017), which requires only minimal changes in the platform. Nevertheless, the current results are in line with a previous report showing reduced responses in the main olfactory bulb during correct rejection trials (Wang et al., 2019). Wang et al. used fiber photometry to measure calcium signals and reported odor-evoked responses during correct rejection trials in both anterior and posterior piriform cortex. However, responses in anterior piriform were comparable with those seen in Hit trials, while posterior piriform responses were smaller. In contrast, our ROI analysis did not reveal significant piriform cortex activation during correct rejection trials. This discrepancy may reflect differences in the spatial specificity of the analysis approach. Future studies may employ modified task structure and more localized analyses to better characterize olfactory system dynamics.

In addition to modifications in experimental design, several technical aspects of the current studies could be improved in future research. First, the current characterization of the HRF is limited by a relatively low temporal resolution (TR = 2.5 s) and the absence of jittering between trial onset and odor onset. Furthermore, the temporal adjacency between different behavioral components (olfactory perception, motor control, and reward processing) complicates the interpretation of the fMRI data. Consequently, it remains unclear whether observed regional differences in the HRF, particularly, arise from low temporal resolution, task structure or true regional variability.

Another technical decision was the use of spin-echo echo-planar imaging (SE-EPI) sequence rather the more common gradient-echo EPI (GE-EPI). While SE-EPI provides lower contrast-to-noise ratio (CNR), it benefits from higher baseline SNR due to the longer T2 relaxation, reduced image distortion, and greater sensitivity to microvasculature, enabling higher spatial specificity at high magnetic fields (S. H. Han et al., 2019; Uludağ et al., 2009; Yacoub et al., 2008). Additionally, the selected TE was relatively short, reducing sensitivity to T2* contrast, but not eliminating it (S. H. Han et al., 2019; Jin et al., 2006; Norris, 2012). This TE was chosen based on prior validation studies linking it to neuronal activity (Kahn et al., 2011, 2013). However, a longer TE and the use of a GE-EPI sequence may yield improved sensitivity.

Despite the putative technical limitations, the resulting fMRI measurement allowed to characterize the functional sub-regions of the mouse striatum. The striatal memory system was preferentially recruited in Hit over FA trials. The striatum is a large structure whose functional specialization is an area of active research (Cox & Witten, 2019; da Silva et al., 2018; Liljeholm & O’Doherty, 2012; Shin et al., 2018; Yartsev et al., 2018). While simultaneous measurement of activity of multiple striatal subregions cannot be easily achieved with optical or electrophysiological methods, our fMRI data lend itself to such investigation, demonstrating differential responses in dorsomedial, dorsolateral, and ventral striatum. This observation is in close agreement with previous reports on striatal division to functional subregions that mediate different types of associations, namely response–outcome, stimulus–response, and stimulus–outcome, respectively (Cox & Witten, 2019; Liljeholm & O’Doherty, 2012). In line with this framework, it was predicted that the dorsal, but not ventral striatum, would be sensitive to motor response variables such as reaction time. Moreover, it was previously shown that dorsolateral, but not dorsomedial striatum, increases its response during late stages of learning (Thorn et al., 2010), a result which was recapitulated in our experiment. The learning-related dynamics we found in the ventral striatum are also consistent with previous reports (Setlow et al., 2003). Overall, we observed that the striatum is driving learning and veridical memory responses in this task.

The hippocampal memory system is known to support associative learning in conditions that depend on single episodes and/or specific contexts, and it has been specifically shown to be involved in odor discrimination (Eichenbaum et al., 1987). In our experiment, the hippocampus showed differential responses in Hit and FA trials, demonstrating negative and positive responses, respectively. Interestingly, the responses in the two conditions were highly correlated, with negative response in Hit trials associated with weaker positive response in FA trials, suggesting that the two memory systems in this task are in competition. Further supporting this interpretation, this response pattern was correlated with shorter reaction times. Finally, FA but not Hit responses showed distinct dynamics over learning, with stronger activations when the animal attained task proficiency, suggesting that in the current task, hippocampal signals emerging at this stage but not during learning drive false memory responses.

Our observations of engagement of striatal learning are overall in agreement with previous reports (Hartley & Burgess, 2005; Iaria et al., 2003; Poldrack et al., 2001; Voermans et al., 2004). However, unlike our results, veridical mnemonic responses in the hippocampus have been previously reported in the context of odor discrimination. Igarashi et al. (2014) demonstrate the coordination of entorhinal–hippocampal ensemble activity during associative learning, revealing a key role for synchronized neuronal activity between these regions in memory formation. Li et al. (2017) expand this by identifying a direct circuit from the entorhinal cortex to hippocampal CA1 that is implicated in olfactory associative learning, suggesting specialized pathways for different sensory modalities. Woods et al. (2020) show that the dentate gyrus classifies cortical representations of learned stimuli, demonstrating its role in processing and differentiating sensory input during learning. Finally, Hassan et al. (2023) explore the hippocampal CA2 region’s role in social odor discrimination, showing how associative learning enhances this process. Together, these studies emphasize the hippocampus’s involvement in processing and learning from sensory and associative cues through distinct regional mechanisms and coordinated activity with the broader hippocampal memory system, focusing on its role in veridical memory responses.

Our results of veridical memory responses associated with striatum but not hippocampal responses diverge from these prior reports. One possibility, is that while hippocampal responses when measured using approaches that allow cellular level characterization identify positive responses, at the population level, these do not drive a hemodynamic response. In parallel, the emergence of hippocampal responses linked to false memory responses is novel. This suggests that here the strong positive responses, assumed to be linked to neural activity in the hippocampus, are what is driving false alarm responses. Consistent with this interpretation, the emergence of false alarms in expert stages highlights potential competition between the systems not observed during task acquisition, highlighting the interactive nature of these memory systems in goal-directed behavior. Similar to the results presented here, several previous studies in rodents showed that information processing mechanisms diverge between the hippocampus and the striatum (van der Meer et al., 2010) and that these systems bidirectionally compete during learning (Lee et al., 2008). This observation was replicated in humans using a feedback-based learning task that was associated with initial hippocampal activation and striatal negative response, but task proficiency results in an inversed response pattern (Poldrack et al., 2001). Moreover, the dissociation between the hippocampus and striatum was also replicated in humans by examining associative learning deficits in clinical populations (Myers et al., 2003). Critically, studies in humans show that under certain conditions, these systems can work in cooperation (Sadeh et al., 2011), but heretofore such interactions were not well characterized in rodents, though several studies described contributions of hippocampus to odor discrimination learning (Hassan et al., 2023; Igarashi et al., 2014; Li et al., 2017; Woods et al., 2020). The current study sets the stage for examination of competition and cooperation between memory systems, and identification of contributions of neural systems that cannot be readily deduced from behavior manipulations alone (Niv, 2021). Identifying homologous findings across species will enable the use of advanced techniques available in mice to examine how chemogenetic or optogenetic control of neural activity affects system dynamics and behavior. Such approach can identify new targets for complementary, cellular-level, electrophysiological, and optical imaging techniques, providing a better understanding of striatal and hippocampal contributions to behavior in health and disease.

Longitudinal task-based fMRI in behaving mice provides a bridge between human and rodent studies, supporting parallel experiments and direct comparisons of the results across species. In the current study, this approach was utilized for examination of the contributions of striatal and hippocampal memory systems during instrumental learning. By employing an imaging technique commonly used in human to rodents, we found similar memory systems dynamics in mice, demonstrating a competitive interaction between the hippocampus and striatum. The mouse striatum drives learning and veridical memory, while the hippocampus contributes to false memory responses, mirroring observations in humans, where the hippocampus and striatum are often seen to compete (Poldrack et al., 2001) or cooperate (Sadeh et al., 2011) depending on the learning context. The use of similar task-based fMRI in behaving rodents and humans bridges methodological gaps (Badre et al., 2015), facilitating translation of findings across species and providing insights into conserved mechanisms of learning and memory.

## Supplementary Material

Supplementary Material

Supplementary Video

## Data Availability

Imaging and behavioral data in this study are available in BIDS format on OpenNeuro, https://openneuro.org/datasets/ds004402. Python code for odor delivery is available at https://github.com/olfa-lab/Voyeur. Code for manuscript is available on OpenNeuro, https://openneuro.org/datasets/ds004402. MRI data were preprocessed using SPM and FSL. Data analysis was conducted in MATLAB (Mathworks).
