# Supplementary material for: Striatal and hippocampal contributions to instrumental learning in the mouse using high-resolution behavioral monitoring and fMRI
Source: Imaging Neurosci (Camb). 2025 Nov 4;3:IMAG.a.975. doi: 10.1162/IMAG.a.975 (PMC12587051; doi:10.1162/IMAG.a.975)
Supplement: Supplementary Material [file IMAG.a.975_supp.pdf]

SUPPLEMENTAL MATERIAL

Striatal and Hippocampal Contributions to Instrumental Learning in the Mouse using High-resolution Behavioral Monitoring and fMRI

Eyal Bergmann,[1,2,] Daniela Lichtman,[1,2] Admir Resulaj,[3,4] Guy Yona,[2] Ornit Nahman,[2] Dmitry Rinberg,[3,5] and Itamar Kahn[1,6,*]

[1]Department of Neuroscience, Mortimer B. Zuckerman Mind Brain Behavior Institute, Columbia University, New York, NY
[2]Department of Neuroscience, Rappaport Faculty of Medicine, Technion – Israel Institute of Technology, Haifa 31096, Israel
[3]Neuroscience Institute, NYU Langone Health, New York, NY
[4]Interdepartmental Neuroscience Program, Northwestern University, Evanston, IL
[5]Center for Neural Science, New York University, New York, NY
[6]Lead contact

*Corresponding Author:
ik2508@columbia.edu
Mortimer B. Zuckerman Mind Brain Behavior Institute
Columbia University
3227 Broadway St., New York, NY 10027

Classification: Psychological and Cognitive Sciences

Keywords: Learning and Memory; Goal-directed behavior; Hippocampus; Striatum; fMRI

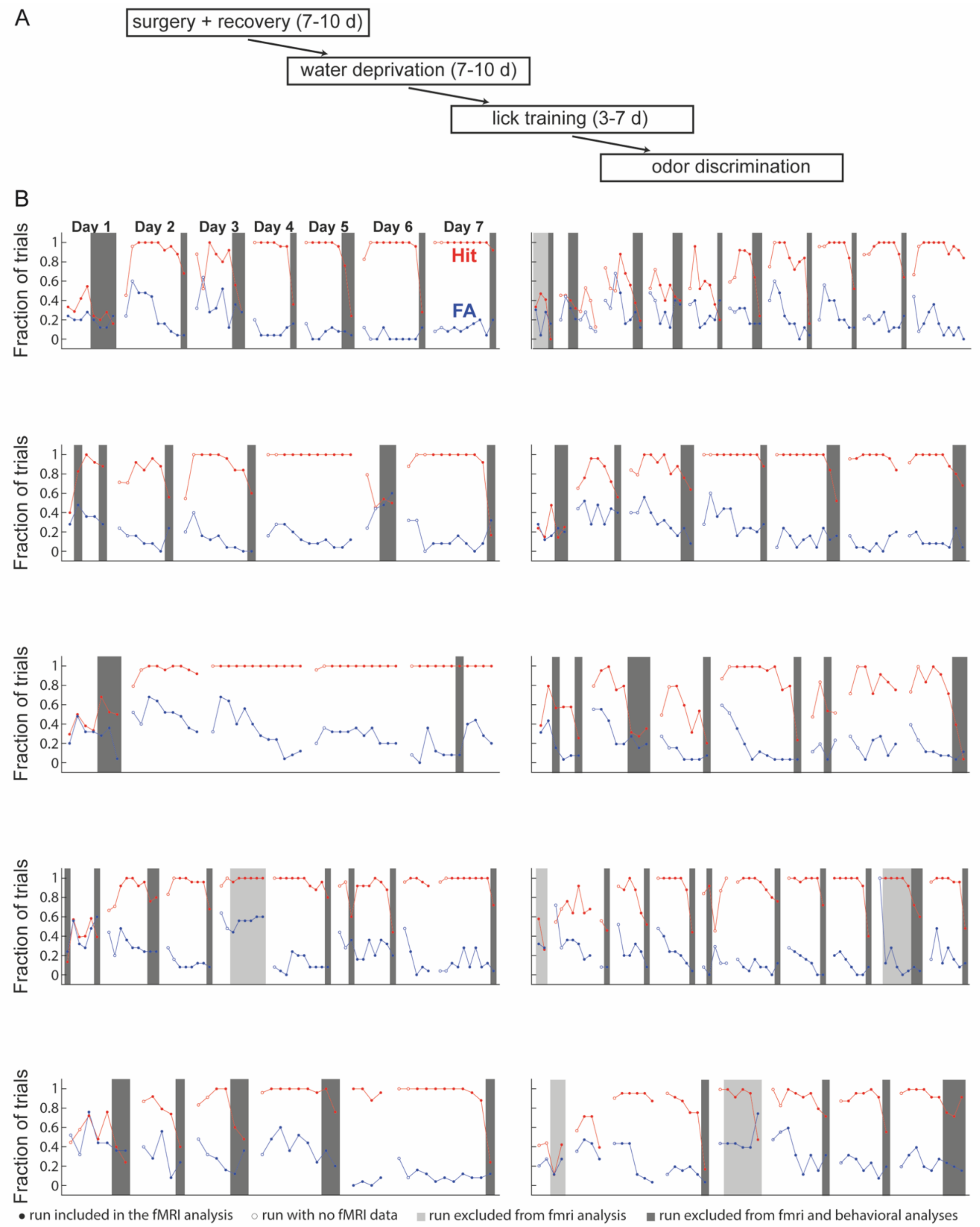

Supplementary Figure 1. Individual learning curves. (A) Preparation and training protocol. (B) The graphs depict the evolution of task performance in individual mice. Each point represents a six min block consisted of 25 trials per odorant. Blocks from the same session are connected. All sessions were included in the behavioral analysis (**Fig. 1**), but some were excluded from the fMRI analysis due to insufficient recording length or dramatic reduction in task performance (see Methods); blocks with no fMRI data (acquired during scanner calibration) were also excluded.

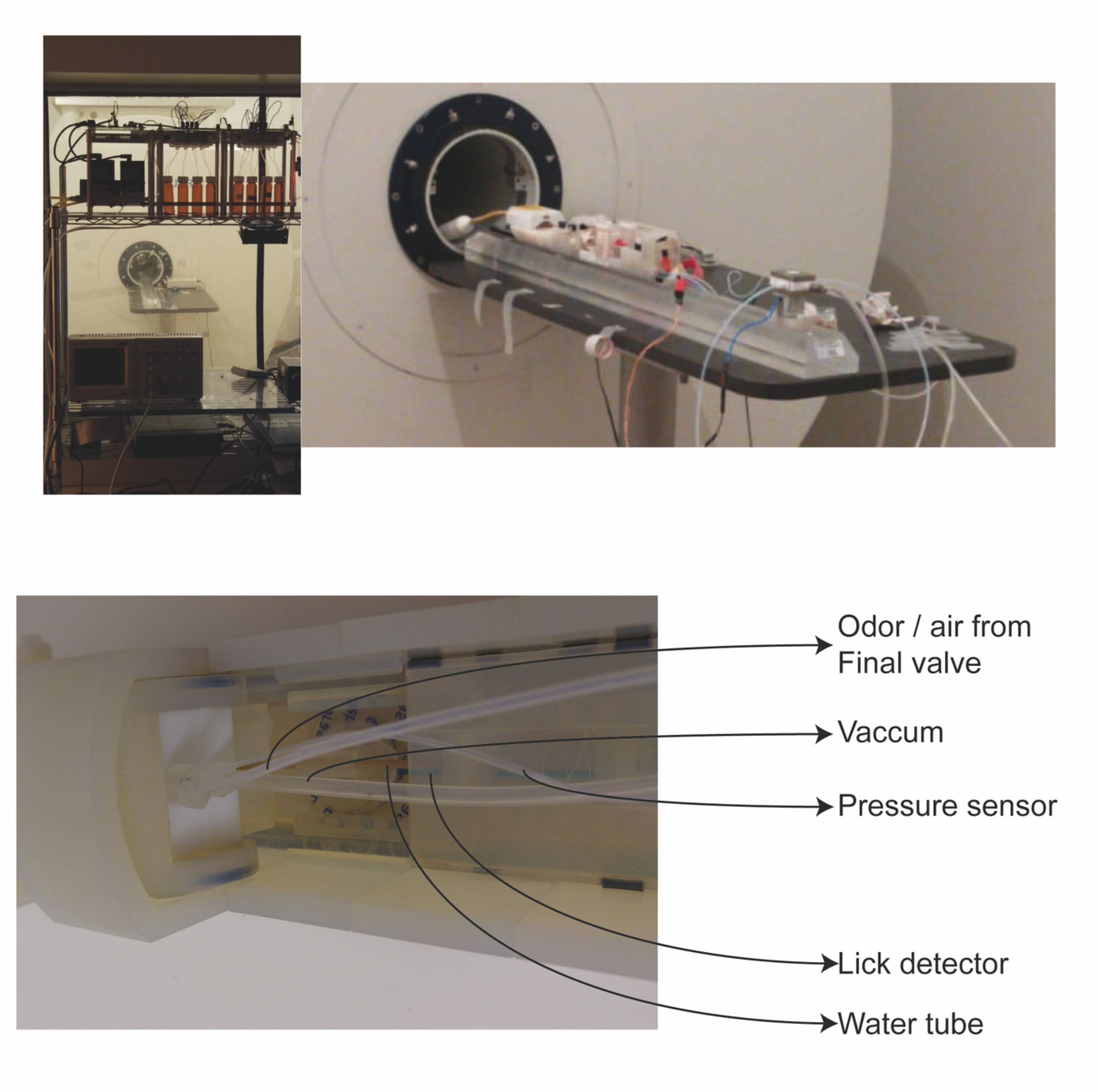

Supplementary Figure 2. Extended experimental setup. Location of olfactometer and final in the MRI room (*top*). A depiction of the posterior aspect of the odor port illustrates the different tubes inserted to the MRI scanner (*bottom*).

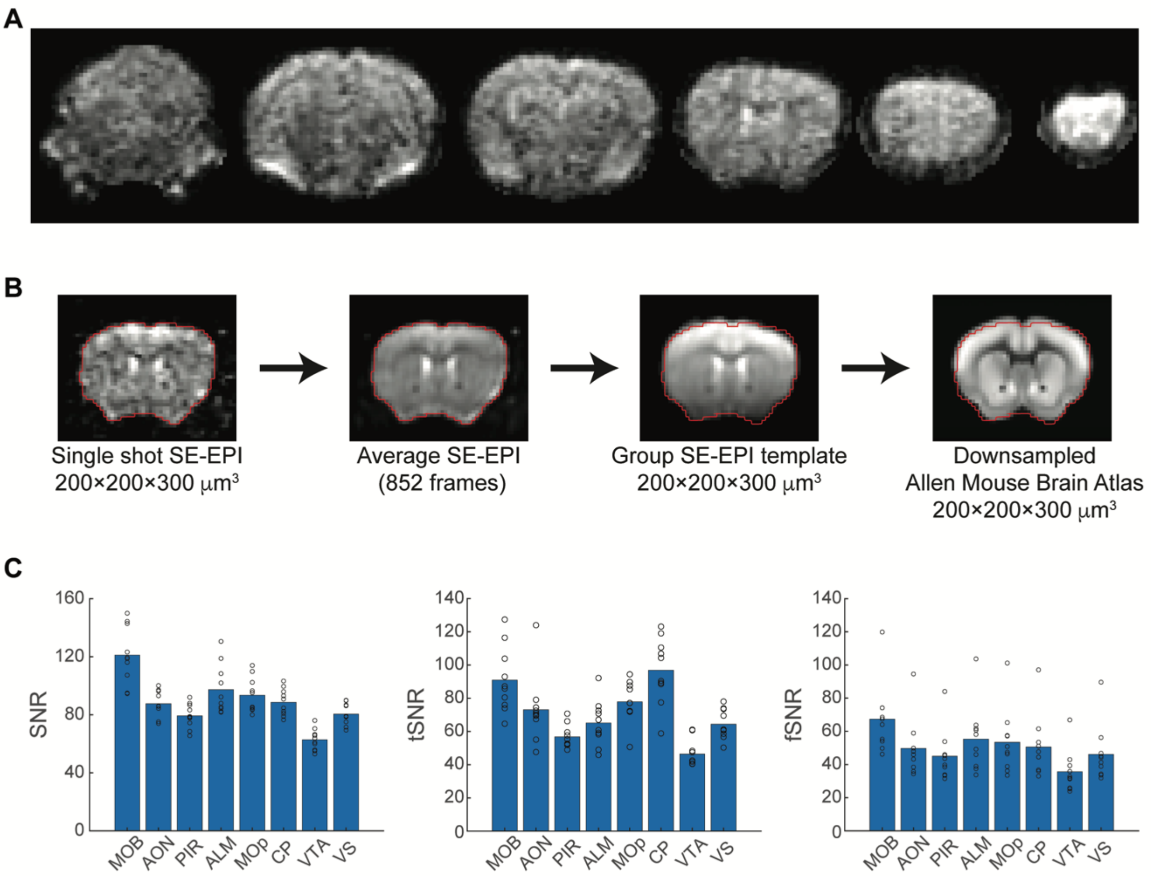

Supplementary Figure 3. Evaluation of MRI signal quality. (A) A single shot EPI image shows preserved image homogeneity despite the addition of behavioral tubes, sensors and actuators to the MRI environment. (**B**) A schematic diagram of the image registration procedure. Single subject average EPI was registered to group average EPI built using a previous resting-state experiment (Bergmann et al., 2016), which itself was normalized to a downsampled version of the Allen Mouse Brain Atlas. The red silhouette depicts the border of the brain in a single session relative to the group template and the histological atlas. (**C**) Signal-to-noise ratio (SNR) measures across brain regions demonstrate adequate and consistent SNR across animals and regions (mean signal in region/standard deviation of signal outside the brain, *left*), temporal SNR (tSNR, mean/standard deviation of signal in region during resting-state, *middle*) and functional SNR (fSNR, mean signal in region in Hit trials/standard deviation of CSF signal in the lateral ventricles during resting-state, *right*). SNR reflects baseline image quality, tSNR reflects temporal stability of the BOLD signal, and fSNR reflects the robustness of task-evoked signals relative to physiological noise. The results show signal attenuation in ventral regions, but still adequate SNR for fMRI analysis. ALM – anterolateral motor cortex, AON – accessory olfactory nucleus, CP – caudoputamen (dorsal striatum), MOB – main olfactory bulb, MOp – primary motor cortex, PIR – piriform areas, VS – ventral striatum (nucleus accumbens), VTA – ventral tegmental area.

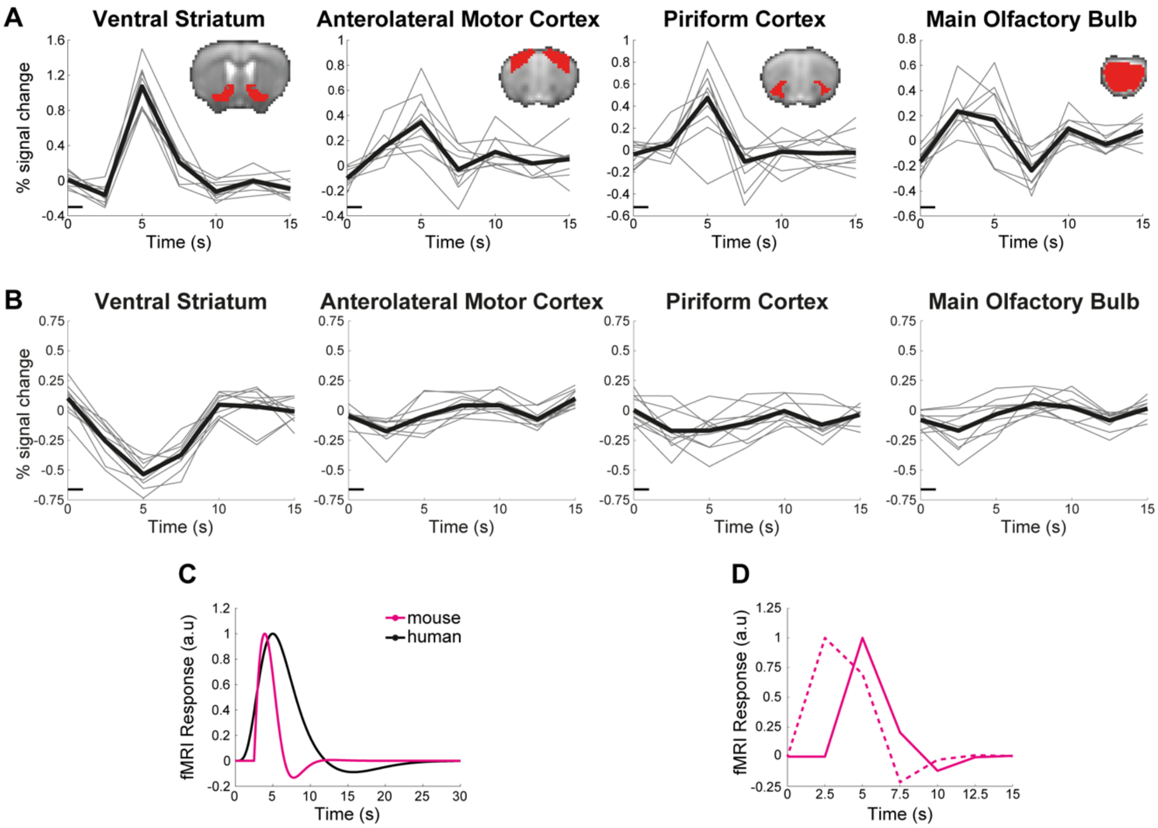

Supplementary Figure 4. Modeling the fMRI hemodynamic response function in awake behaving mice. (**A**) Hemodynamic responses for correct 'go' trials (Hit) extracted from pre-defined regions in the Allen Mouse Brain Atlas demonstrate reliable responses in main olfactory bulb, piriform cortex, anterolateral motor cortex and ventral striatum with distinct temporal dynamics. (**B**) Replication of the ROI analysis of the pre-defined labels from the Allen Mouse Brain Atlas for the Correct Rejection condition demonstrates null or negative responses in main olfactory bulb, piriform cortex, anterolateral motor cortex and ventral striatum. (**C**) Modelling of the hemodynamic response function (HRF) in the behaving mouse based on the ventral striatum ROI revealed shorter rise time and response duration compared to the canonical HRF used in humans. (**D**) temporal convolution of the modeled HRF a stick function resulted in a main response regressor at fMRI resolution. A shifted version of this response (dashed line) was used to capture responses in the main olfactory bulb. The black line represents the duration of the olfactory stimulus.

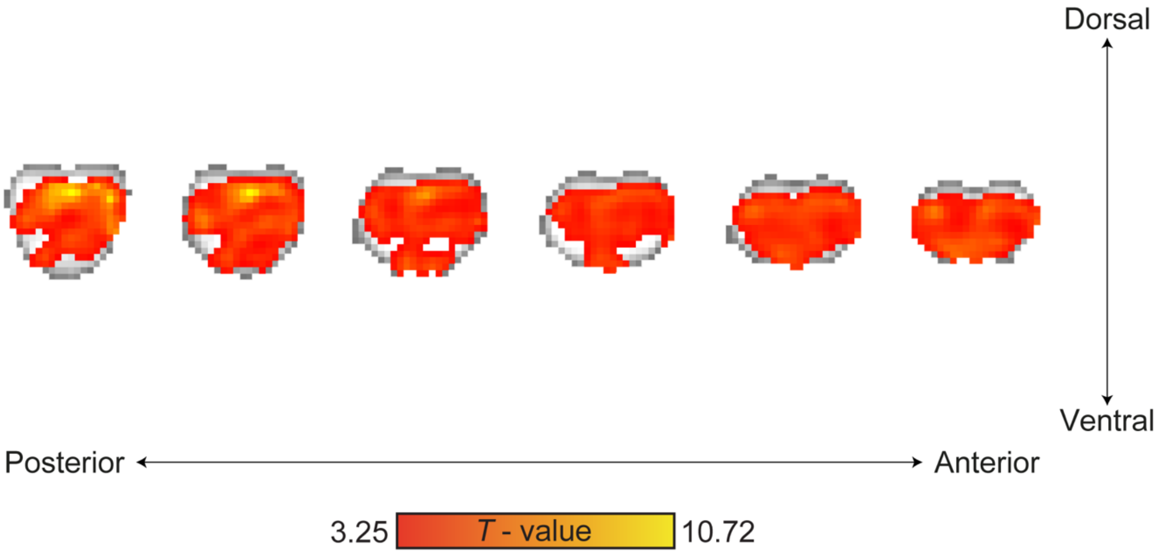

Supplementary Figure 5. fMRI hemodynamic responses in the main olfactory bulb. A statistical parametric map (n = 10) of Hit > baseline contrast using a shifted HRF showing distributed voxel-wise responses in the main olfactory bulb. The map is presented on an average raw fMRI data (P < 0.05, corrected for multiple comparisons using false-discovery rate correction, voxel extent of 5.

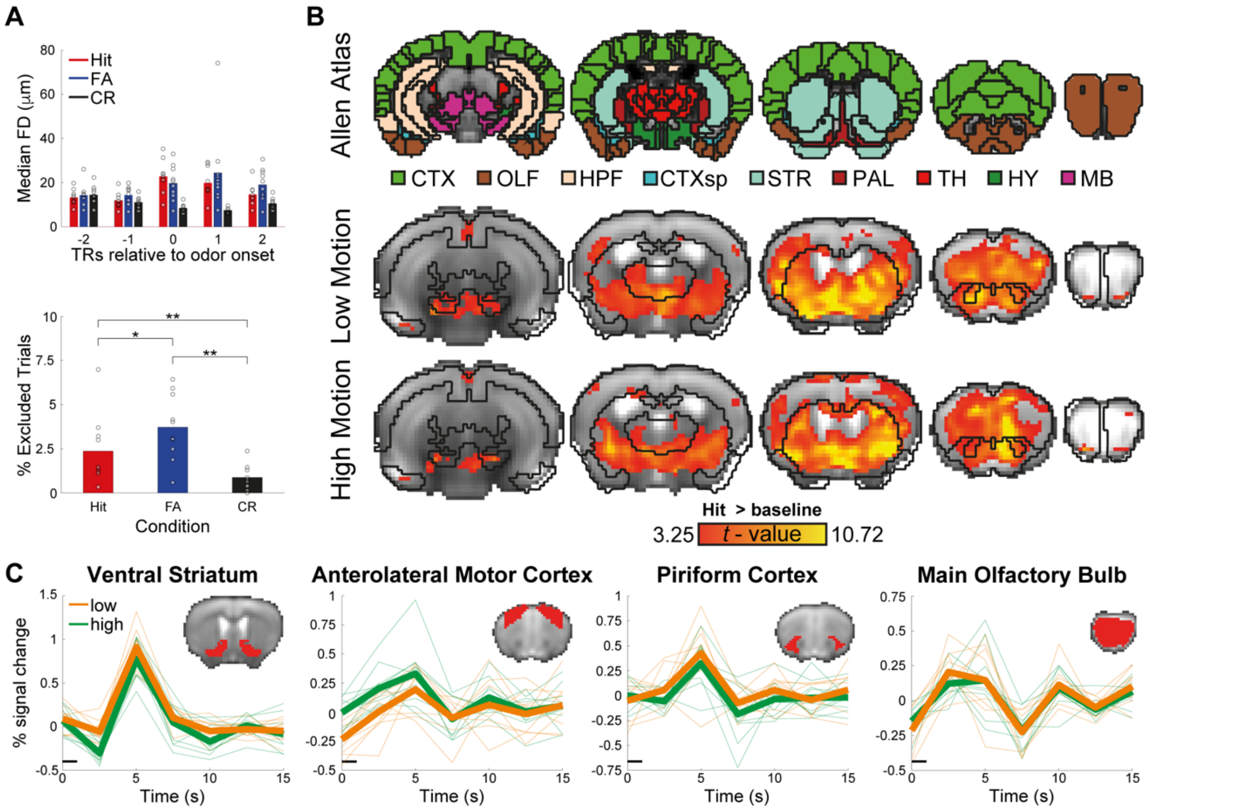

Supplementary Figure 6. Effects of head motion on task-evoked responses. (**A**) Median framewise displacement before and after Hit, FA and CR trials (*top*) demonstrates similar baseline motion with differential changes during task with increased head motion in Hit and FA trials and decreased motion in CR trials as measured by a significant interaction between time condition using a repeated-measures ANOVA (Hit vs CR: $F_{(4, 36)} = 49.5$, $P < 0.001$, $\varepsilon_{H\text{-}F} = 0.587$, FA vs. CR: $F_{(4, 36)} = 6.99$, $P < 0.001$, $\varepsilon_{H\text{-}F} = 0.373$; Hit vs. FA: $F_{(4, 36)} = 1.76$, $P = 0.21$, $\varepsilon_{H\text{-}F} = 0.396$). Comparison between the fraction of excluded trials (frames displacement > 200 μm) in different conditions (*bottom*) reveal increased exclusion in FA trials, followed by Hit and then CR trials (two-tailed paired Student's t-test: Hit vs. CR: $t_{(9)} = 3.55$, $P = 0.006$, FA vs. CR: $t_{(9)} = 6.17$, $P < 0.001$, FA vs. Hit: $t_{(9)} = 3.63$, $P = 0.005$). (**B**) All Hit trials were divided to three thirds based on framewise displacement values of the first frame after odor onset. Statistical maps were created for the high (top 33%) and low (bottom 33%) framewise displacement values to characterize motion-related artifacts. Yet, the analysis reveal that the two maps demonstrate high overlap (Sørensen–Dice coefficient = 0.823). (**C**) Hemodynamic responses for Hit trials with high (top 33%) and low (bottom 33%) head motion extracted from pre-defined regions in the Allen Mouse Brain Atlas demonstrate similar responses in main olfactory bulb, piriform cortex, anterolateral motor cortex and ventral striatum; the shaded areas depict the standard error of the mean.

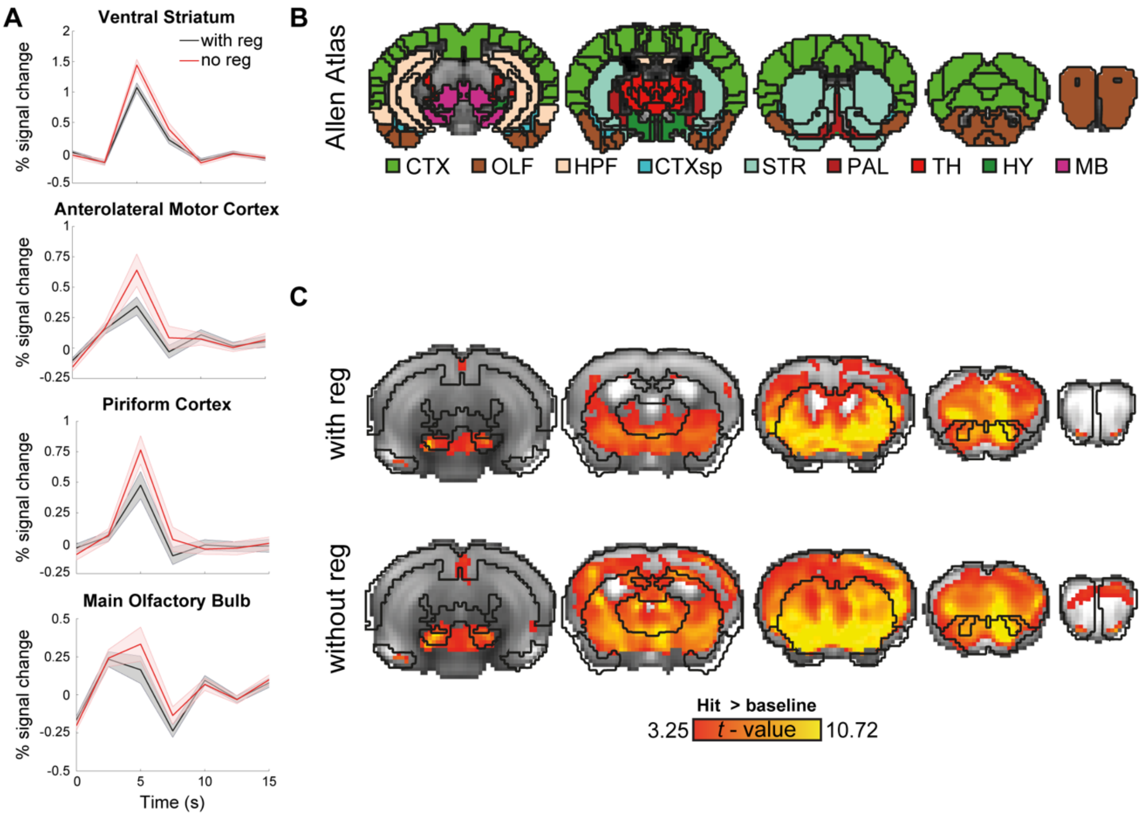

Supplementary Figure 7. Effects of CSF and global signals nuisance regressors on task-evoked responses. (**A**) Hemodynamic responses for correct 'go' trials (Hit) extracted from pre-defined regions in the Allen Mouse Brain Atlas before (red) and after (black) the addition of CSF and global signals and their derivatives as nuisance regressors to the GLM; the shaded areas depict the standard error of the mean. (**B**) Allen Mouse Brain Atlas labels were overlaid on average raw fMRI data (spin-echo echo planar imaging). CTX – cerebral cortex; CTXsp – cortical subplate; HPF – hippocampal formation; HY – hypothalamus; MB – midbrain; OLF – olfactory areas; PAL – pallidum; STR – striatum; TH – thalamus. (**C**) Group statistical parametric maps generated with (*top*) and without (*bottom*) CSF and global signals and their derivatives as nuisance regressors in the GLM. While the maps are generally similar (Sørensen–Dice coefficient = 0.79), CSF and global signal regression is associated with reduced response in somatosensory and motor cortices.

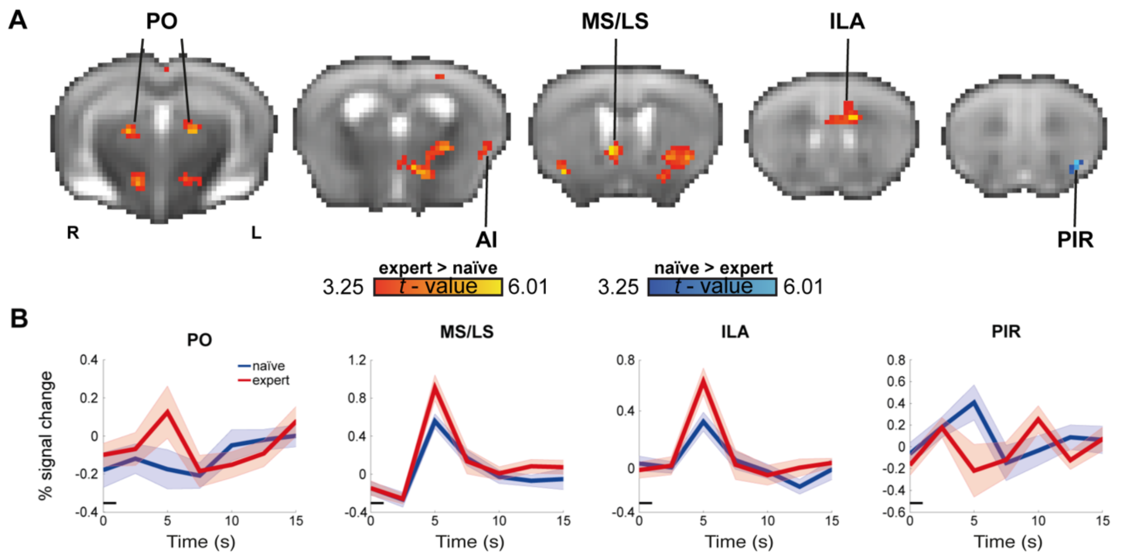

Supplementary Figure 8. The effects of task proficiency on brain responses in the odor discrimination task. (**A**) Statistical parametric maps of paired comparisons between expert > naïve (*red-yellow*) and naïve > expert (*blue-light blue*) contrasts for Hit trials; the maps are presented on an average raw fMRI data (spin-echo echo planar imaging in radiological convention) and annotated based on labels from the Allen Mouse Brain Atlas (AI – agranular insular area; ILA – infralimbic area; MS/LS – medial/lateral septal complex, PIR – piriform area; PO – posterior complex of the thalamus; STR – striatum (caudoputman/nucleus accumbens); $P < 0.005$, uncorrected for multiple comparisons. (**B**) ROI analysis of Hit responses in clusters identified using a GLM in naïve (first ten blocks) and expert (last ten blocks) stages. The black line depicts the onset of the odor stimulus relative to the hemodynamic response; the shaded areas depict the standard error of the mean.

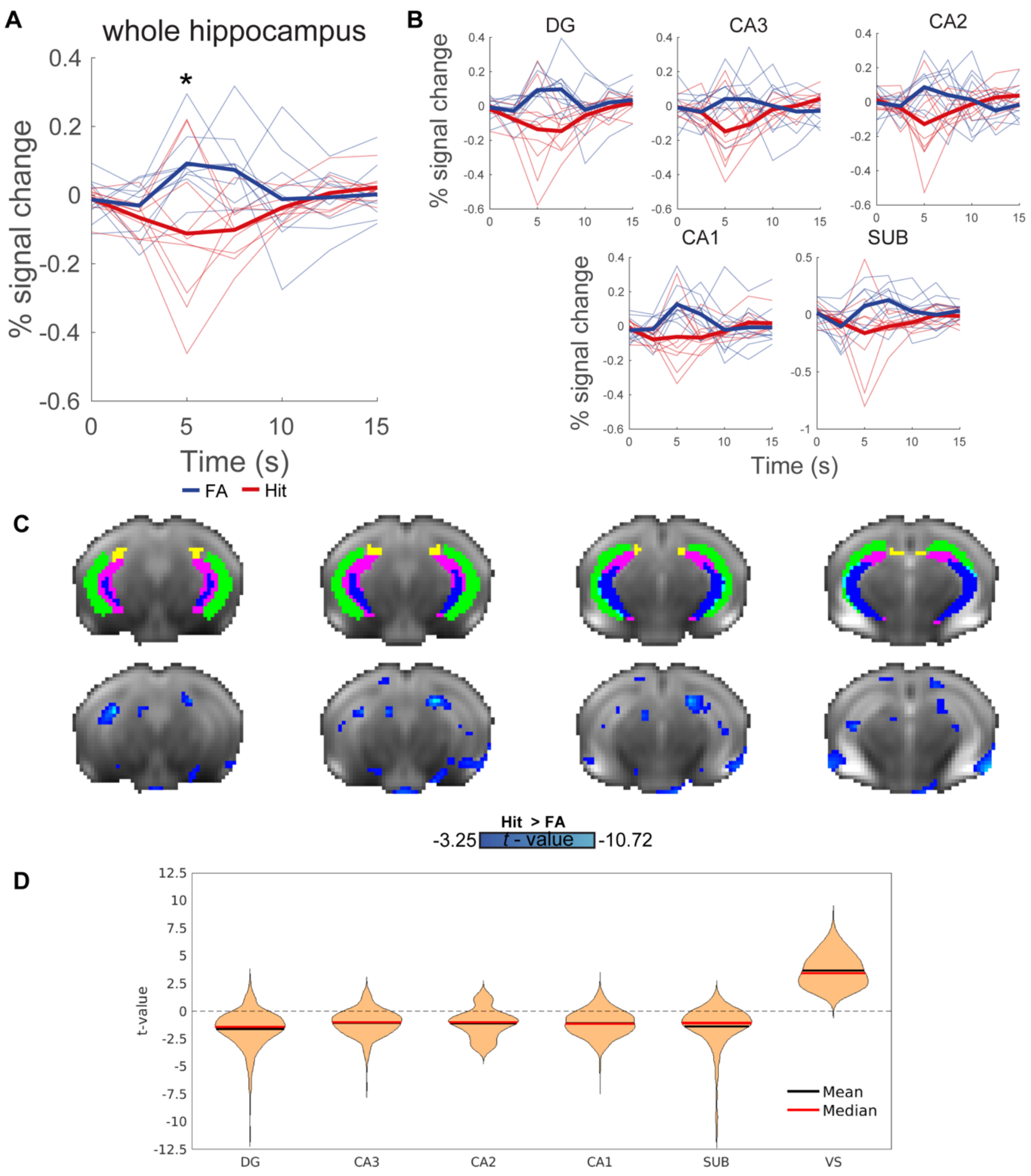

Supplementary Figure 9. Subfield analysis of hippocampal response. (**A**) ROI analysis of a whole hippocampal ROI demonstrates significant activation in FA trials ($t_{(9)} = 3.26$, P = 0.01), as well as preferential response in FA over Hit trials ($t_{(9)} = 2.44$, P = 0.037). (**B**) ROI analysis of different hippocampal subfields demonstrates consistent responses across subfields (repeated-measures ANOVA: condition: $F_{(1,9)} = 6.03$, P = 0.036; subfield: $F_{(4,36)} = 1.94$, P = 0.124; condition × subfield: $F_{(4,36)} = 0.16$ P = 0.96). (**C**) A negative statistical parametric map of Hit > FA contrast reveals at a lenient threshold (P < 0.005, uncorrected for multiple comparisons, voxel extent of 5). Subfields are marked as follows: dentate gyrus (DG, magenta), CA3 (blue), CA2 (light blue), CA1 (green) and subiculum (SUB, yellow). (**D**) distribution of t-values of Hit > FA contrast demonstrates consistent pattern across hippocampal subfields as opposed to the ventral striatum (VS).

Supplementary Video 1.

A mouse performing the task after reaching criterion. Dynamic annotations of the experimental apparatus, as well as measured non-invasive sniff detection, lick responses, and trials are shown. *Top*: A head-fixed mouse is shown with a single-element RF 20 mm loop coil. *Middle*: A line-plot of the lick pressure sensor connected to the water spout is shown. When the mouse touches the spout, the pressure sensor is showing a decrease in signal. Ticks in *black* denote detected licks (decreases in the lick signal), and water reward dispensation is shown below in *blue*. *Bottom*: The sniff signal is shown oscillating between inhalation and exhalation. Go odors are denoted in *green* and no-go odors in *red*. A sample frame is shown here. *Duration*: 254 s.

Supplementary Table 1

List of brain regions with significant response in correct Hit over baseline contrast (P < 0.05, corrected for multiple comparisons using the false-discovery rate method).

Structure	Left Hemisphere (voxels)	Right Hemisphere (voxels)
**Isocortex**		
Frontal pole, cerebral cortex	30	10
Primary motor area	160	113
Secondary motor area	228	121
Primary somatosensory area, nose	23	68
Primary somatosensory area, barrel field	0	12
Primary somatosensory area, mouth	154	124
Primary somatosensory area, upper limb	9	30
Primary somatosensory area, unassigned	0	11
Supplemental somatosensory area	63	71
Gustatory areas	39	42
Visceral area	5	8
Anterior cingulate area	93	45
Prelimbic area	79	59
Infralimbic area	33	24
Orbital area	215	179
Agranular insular area	132	135
Retrosplenial area	6	0
**Olfactory areas**		
Main olfactory bulb	63	67
Anterior olfactory nucleus	132	107
Taenia tecta	27	22
Dorsal peduncular area	22	20
Piriform area	63	93
Cortical amygdalar area	0	14
Postpiriform transition area	0	5
**Hippocampal formation**		
Field CA1	0	6
Field CA3	0	11
Dentate gyrus	7	10
**Cortical subplate**		
Claustrum	18	18
Endopiriform nucleus	35	42
**Striatum**		
Caudoputamen	681	665

Nucleus accumbens	170	192
Fundus of striatum	17	17
Olfactory tubercle	11	9
Lateral septal nucleus	45	59
Anterior amygdalar area	14	12
Central amygdalar nucleus	34	20
Medial amygdalar nucleus	19	14
**Pallidum**		
Globus pallidus, external segment	50	47
Globus pallidus, internal segment	19	12
Substantia innominata	99	103
Magnocellular nucleus	11	12
Medial septal complex	20	13
Triangular nucleus of septum	6	0
Bed nuclei of the stria terminalis	38	26
**Thalamus**		
Ventral group of the dorsal thalamus	39	44
Ventral posteromedial nucleus of the thalamus	0	12
Ventral posteromedial nucleus of the thalamus, parvicellular part	6	0
Lateral group of the dorsal thalamus	0	9
Anterior group of the dorsal thalamus	7	11
Anteromedial nucleus	14	6
Medial group of the dorsal thalamus	23	22
Midline group of the dorsal thalamus	12	11
Intralaminar nuclei of the dorsal thalamus	12	0
Reticular nucleus of the thalamus	15	17
**Hypothalamus**		
Periventricular zone	7	6
Periventricular region	58	44
Hypothalamic medial zone	80	61
Hypothalamic lateral zone	126	121
**Midbrain**		
Substantia nigra, reticular part	13	17
Ventral tegmental area	14	7

Supplementary Table 2
List of brain regions with significant preference in the Hit vs. False Alarm contrast (P < 0.05, corrected for multiple comparisons using the false-discovery rate method).

Hit > FA

Structure	Left Hemisphere (voxels)	Right Hemisphere (voxels)
**Isocortex**		
Frontal pole, cerebral cortex	5	0
Primary motor area	10	5
Secondary motor area	9	0
Supplemental somatosensory area	8	7
Gustatory areas	0	11
Visceral area	0	7
Agranular insular area	16	19
**Olfactory areas**		
Main olfactory bulb	27	11
Anterior olfactory nucleus	11	16
Taenia tecta	0	9
Piriform area	12	45
**Cortical subplate**		
Claustrum	8	0
Endopiriform nucleus	10	22
**Striatum**		
Caudoputamen	106	91
Nucleus accumbens	63	97
Fundus of striatum	0	14
Olfactory tubercle	9	0
Lateral septal nucleus	0	8
**Pallidum**		
Substantia innominata	22	33
Magnocellular nucleus	0	7
Medial septal complex	9	11
**Hypothalamus**		
Periventricular region	21	0
Hypothalamic medial zone	19	0
Hypothalamic lateral zone	21	12

FA > Hit

Structure	Left Hemisphere (voxels)	Right Hemisphere (voxels)
**Olfactory areas**		
Piriform area	21	10
**Hippocampal formation**		
Dentate gyrus	5	7
Entorhinal area, lateral part	6	0

Supplementary Table 3
ROI analysis of brain responses to Hit and FA trials for clusters

Region	Hit (mean±SD)	FA (mean±SD)	t-value	p-value	Effect size
MOp	0.59±0.17%	0.02±0.24%	$t_{(9)} = 7.46$	P < 0.001	d = 2.36
AI	0.77±0.17%,	0.07±0.15%,	$t_{(9)} = 8.34$	P < 0.001	d = 2.64
LHA	0.63±0.18%	0.05±0.09%	$t_{(9)} = 10$	P < 0.001	d = 3.16
PVH	1±0.32%	0.14±0.16%	$t_{(9)} = 6.38$	P < 0.001	d = 2.02
STR	0.94±0.21%,	0.16±0.14%	$t_{(9)} = 9.35$	P < 0.001	d = 2.96
HC	-0.37±0.29%,	0.2±0.23%,	$t_{(9)} = -11.92$	P < 0.001	d = -3.77
